# Changes in morphogen kinetics and pollen grain size are potential mechanisms of aberrant pollen aperture patterning in previously observed and novel mutants of *Arabidopsis thaliana*

**DOI:** 10.1371/journal.pcbi.1006800

**Published:** 2019-02-28

**Authors:** Shayne M. Plourde, Prativa Amom, Michelle Tan, Adriana T. Dawes, Anna A. Dobritsa

**Affiliations:** 1 Department of Mathematics, The Ohio State University, Columbus, Ohio, United States of America; 2 Department of Molecular Genetics, The Ohio State University, Columbus, Ohio, United States of America; University of California Irvine, UNITED STATES

## Abstract

Pollen provides an excellent system to study pattern formation at the single-cell level. Pollen surface is covered by the pollen wall exine, whose deposition is excluded from certain surface areas, the apertures, which vary between the species in their numbers, positions, and morphology. What determines aperture patterns is not understood. *Arabidopsis thaliana* normally develops three apertures, equally spaced along the pollen equator. However, Arabidopsis mutants whose pollen has higher ploidy and larger volume develop four or more apertures. To explore possible mechanisms responsible for aperture patterning, we developed a mathematical model based on the Gierer-Meinhardt system of equations. This model was able to recapitulate aperture patterns observed in the wild-type and higher-ploidy pollen. We then used this model to further explore geometric and kinetic factors that may influence aperture patterns and found that pollen size, as well as certain kinetic parameters, like diffusion and decay of morphogens, could play a role in formation of aperture patterns. In conjunction with mathematical modeling, we also performed a forward genetic screen in Arabidopsis and discovered two mutants with aperture patterns that had not been previously observed in this species but were predicted by our model. The *macaron* mutant develops a single ring-like aperture, matching the unusual ring-like pattern produced by the model. The *doughnut* mutant forms two pore-like apertures at the poles of the pollen grain. Further tests on these novel mutants, motivated by the modeling results, suggested the existence of an area of inhibition around apertures that prevents formation of additional apertures in their vicinity. This work demonstrates the ability of the theoretical model to help focus experimental efforts and to provide fundamental insights into an important biological process.

## Author summary

Pollen is renowned for its ability to form beautiful and complex patterns on its surface. One of the most prominent patterns on the pollen surface is formed by apertures, the regions that lack deposition of the pollen wall exine and develop at precise locations which often vary between the species. How aperture patterns are created is an intriguing and poorly understood question. We developed a mathematical model that aims to explore the mechanisms responsible for the aperture patterning in the pollen of the model plant Arabidopsis. Our model showed that size of the pollen grain could be solely responsible for the increase in aperture number observed in the pollen of some Arabidopsis mutants. Additionally, kinetic parameters, such as diffusion and decay of aperture factors, could also influence aperture number. We coupled our mathematical modeling with a forward genetic screen of a mutagenized population of Arabidopsis. This screen discovered novel mutants with aperture patterns that had been predicted by our mathematical model. Further experiments on these mutants provided additional support to the modeling predictions. These results demonstrate that mathematical modeling could be a powerful tool for understanding the mechanisms responsible for patterning of pollen grains.

## Introduction

The process of cell morphogenesis often depends on the ability of cells to form distinct domains of plasma membrane and precisely target deposition of extracellular materials. Pollen presents a powerful model to study the mechanisms that control formation of membrane domains and localization of extracellular structures. Pollen grains are surrounded by a complex extracellular structure, exine, that can assemble into thousands of elaborate, species-specific patterns, which make the pollen surface one of the most diverse structures found in nature [1–3]. These patterns are formed by precisely depositing exine at certain areas of the pollen surface and by preventing or reducing its deposition at other sites.

**Fig 1:**
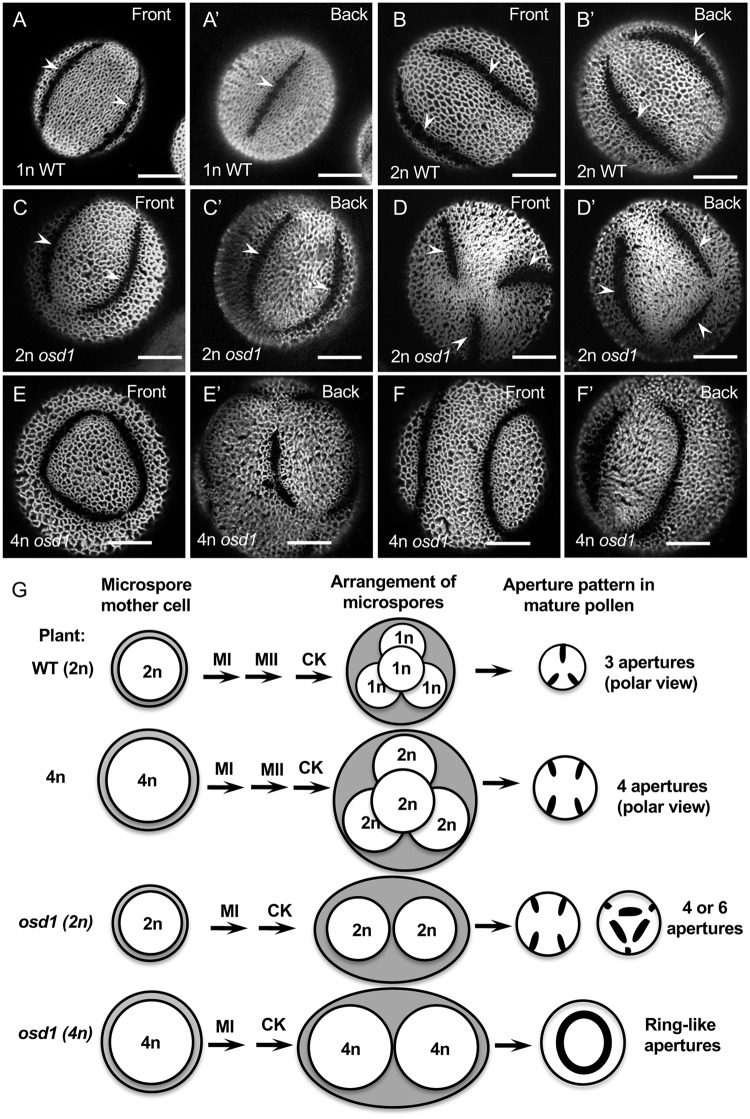
Changes in pollen ploidy and size correlate with changes in aperture number. (A-A’) Haploid pollen of wild-type Arabidopsis (Landsberg *erecta* ecotype) have three apertures (arrowheads). Front and back views of the auramine O-stained pollen grains are shown here and in other images as indicated. (B-B’) Diploid Landsberg pollen grains from tetraploid plants are larger than haploid pollen grains and have primarily four apertures (arrowheads). (C-D’) Diploid pollen grains from a diploid *osd1* mutant usually have either four (C-C’) or six (D-D’) apertures (arrowheads). (E-F’) Irregular aperture patterns, often adopting a ring-shaped morphology, are common among tetraploid pollen grains from the tetraploid *osd1* mutants. (G) A diagram of pollen development and aperture formation in the wild-type Arabidopsis and in several mutants with abnormal pollen ploidy. The ploidy of microspore mother cells (MMC), which divide meiotically to generate microspores, is the same as in the plants producing these MMCs. In the wild-type (WT) Arabidopsis, the 2n MMC undergoes two rounds of nuclear division (MI and MII), followed by cytokinesis (CK), and produces a tetrad of 1n microspores arranged in the tetrahedral conformation. The grey areas around the MMCs and the postmeiotic microspore arrangements represent the callose walls. First signs of apertures become apparent in postmeiotic microspores held together by the callose wall. After the callose wall dissolution, each microspore develops into a mature pollen grain, which, depending on its ploidy, exhibits a characteristic number of apertures. In the *osd1* mutant, the second nuclear division (MII) is skipped, resulting in the formation of a dyad of microspores whose ploidy is identical to that of their MMC precursor. Scale bars = 10 *μ*m.

In most plant species the restriction of exine deposition at particular locations leads to the development of some of the most obvious patterning elements on the pollen surface. These characteristic areas which either lack exine completely or have decreased amounts of exine are called apertures [2, 4, 5] (Fig 1). Apertures are critical for pollen viability and function, as they often serve as portals through which pollen tubes exit during germination and as architectural details that help the pollen accommodate volume changes in response to changing hydration levels [5–9].

The patterns produced by apertures on the pollen surface are one of the major taxonomic features used for classification of flowering plants. The two major clades of flowering plants, eudicots and monocots, are each characterized by a prototypical aperture pattern, defined by aperture number and position. Monocots tend to have a single, polarly localized aperture, whereas eudicots most commonly have three apertures, equidistantly distributed like three meridians around the pollen equator. Although these are the most prevalent patterns for these clades, individual species exhibit wide variations on these common themes, with dramatic differences in aperture number, position, and morphology. Among the observed patterns are pore-like single apertures in grasses, two hole-like apertures at the opposite poles in many species of bromeliads, three equatorial apertures in tomato, four equatorial apertures in some species of tobacco, six apertures in species of mint and passion flower, multiple hole-like apertures in hibiscus and phlox, and many other possible patterns [1, 2, 4, 5]. In the cases when pollen has more than one aperture, these apertures (or their centers) tend to be equally distributed on the pollen surface or around the pollen equator. Yet, the molecular and cellular mechanisms that restrict exine deposition at these specific sites and contribute to the formation of aperture patterns are not understood in any species.

In the model plant *Arabidopsis thaliana*, like in many other eudicot species, pollen has three long and narrow apertures placed equidistantly around the equator of the pollen grains (Fig 1A, 1A’ and 1G). This patterning is very precise: essentially all pollen grains in the wild-type Arabidopsis develop this pattern. Formation of Arabidopsis apertures begins after male meiosis, when four meiotic products—the pollen precursors known as the microspores—are held together in a tetrad arrangement, surrounded and separated from each other by a callose wall [10–12] (Fig 1G).

Until recently, only a single molecular player, the product of the Arabidopsis *INAPERTURATE POLLEN1* (*INP1*) gene, was known to act as an aperture-promoting factor. During the tetrad stage INP1 protein specifically aggregates at the areas of the microspore plasma membrane that will become apertures, while in the absence of functional INP1 apertures do not form [12, 13]. Although INP1 is likely a late-acting factor which does not by itself define the aperture pattern [14, 15], its specific localization to certain membrane sites suggests that the plasma membrane in microspores acquires polarity and forms distinct domains, which then become protected from exine deposition. Recently, we identified a second aperture factor in Arabidopsis, the protein kinase D6 PROTEIN KINASE-LIKE3 (D6PKL3), which appears to act upstream of INP1 and helps attract it to the aperture domains [16]. However, the mechanism by which these proteins select domains for aperture sites is not understood.

Previously, we demonstrated that the mechanism of aperture formation is sensitive to pollen ploidy or to factors tightly linked to ploidy, such as pollen size [13]. Compared to the normal haploid (1n) pollen grains in Arabidopsis with three equatorial apertures, the larger diploid (2n) pollen grains generated by several mechanisms (e.g. through a tetrad or dyad stage) commonly develop either four equatorial apertures or six apertures distributed along the edges of a tetrahedron (Fig 1B–1D’ and 1G), although some other patterns are also observed [13]. In turn, pollen of even higher ploidy (3n or 4n) develops complex aperture patterns with the larger number of apertures that often coalesce into ring-shaped structures (Fig 1E–1G) [13]. INP1 localization in higher-ploidy microspores recapitulates the changed number of apertures in the mature pollen [13, 14], suggesting that the number and placement of membrane aperture domains changes in these cells. However, it is not known what factors linked to ploidy (for example, biochemical—e.g. gene dosage and levels of gene expression, or mechanical—e.g. pollen size) are responsible for changes in membrane domains and aperture number [13, 17, 18].

In addition to pollen ploidy, factors connected to male meiosis and meiotic cytokinesis have been hypothesized to be involved in aperture formation [5, 10, 19–24]. In eudicot species which often have a tetrahedral arrangement of microspores in a tetrad and form three apertures on each microspore, these apertures tend to develop at the centers of the six centripetally growing division plates, close to the three points of last contact that each microspore has with its three sisters at the end of meiotic cytokinesis [5, 10, 24]. These observations led to the hypothesis that points of last contact may serve as patterning landmarks for aperture positions [5, 10, 24]. Although our recent results indicated that the points of last contact *per se* are unlikely to act as the determinants for aperture placement [13], the strong correlation between their positions and positions of apertures suggest a possibility that meiotic cytokinesis may provide some prepatterning cues for aperture sites.

**Fig 2:**
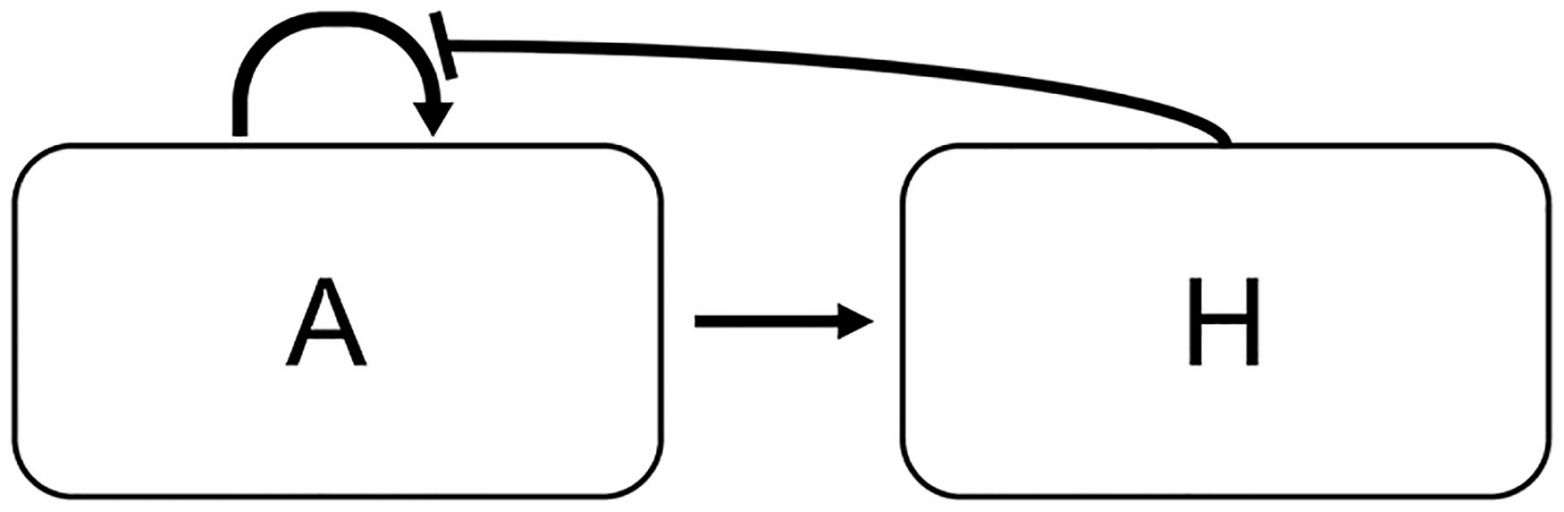
Morphogen interaction diagram. The activator (A) self-activates and activates the inhibitor. The inhibitor (H) inhibits the activator.

To explore biochemical and geometric parameters that may be responsible for the observed aperture patterns but cannot yet be approached experimentally, we turned to mathematical modeling. As with other studies where little is known about the underlying interaction network [25], a pattern-forming model can be used to study broad properties of the biological system. The equally spaced distribution of the INP1-decorated membrane domains and apertures in Arabidopsis is reminiscent of patterns that can be generated with mathematical pattern-formation models. A simple reaction-diffusion system, first introduced by Alan Turing [26] and later developed into the Gierer-Meinhardt (GM) system of equations [27, 28], which simulates the interaction of two morphogens, a short-range activator and a long-range inhibitor (Fig 2), was used as the basis for our model. The GM model was chosen because it had been previously used to successfully model a variety of patterns, including some similar to aperture patterns [28–30]. Furthermore, a Turing-type model has been previously used to investigate pattern formation on the surface of a sphere, like pollen [31], although the patterns matching pollen aperture number and placement have not been explored in previous studies.

Here we focused on the specification of aperture number and placement of aperture centers on a single pollen grain. A deterministic model with stochastic initial conditions was able to reliably produce the three-aperture pattern characteristic of the wild-type Arabidopsis pollen. After the model was parametrized, we tested different variables that may have an effect on the number and positions of apertures, such as domain size, morphogen kinetics, initial conditions, and possible pre-patterns.

We found that the domain size and morphogen kinetics have the largest impact on the aperture patterns produced by our simulations. In addition, in parallel with modeling, we performed a new forward genetic screen in Arabidopsis and have identified novel aperture mutants. These mutants develop phenotypes that have not been previously observed in this species but were predicted by our model. We have then used these mutants to further test the model predictions by increasing ploidy of the mutant pollen. Through integration of computational and experimental approaches we have extended the framework for the process of pollen aperture formation and provided insight into the potential causes of mutant pollen patterning.

## Results

### The Gierer-Meinhardt model is able to recapitulate aperture number and patterns in 1D and 3D domains

**Fig 3:**
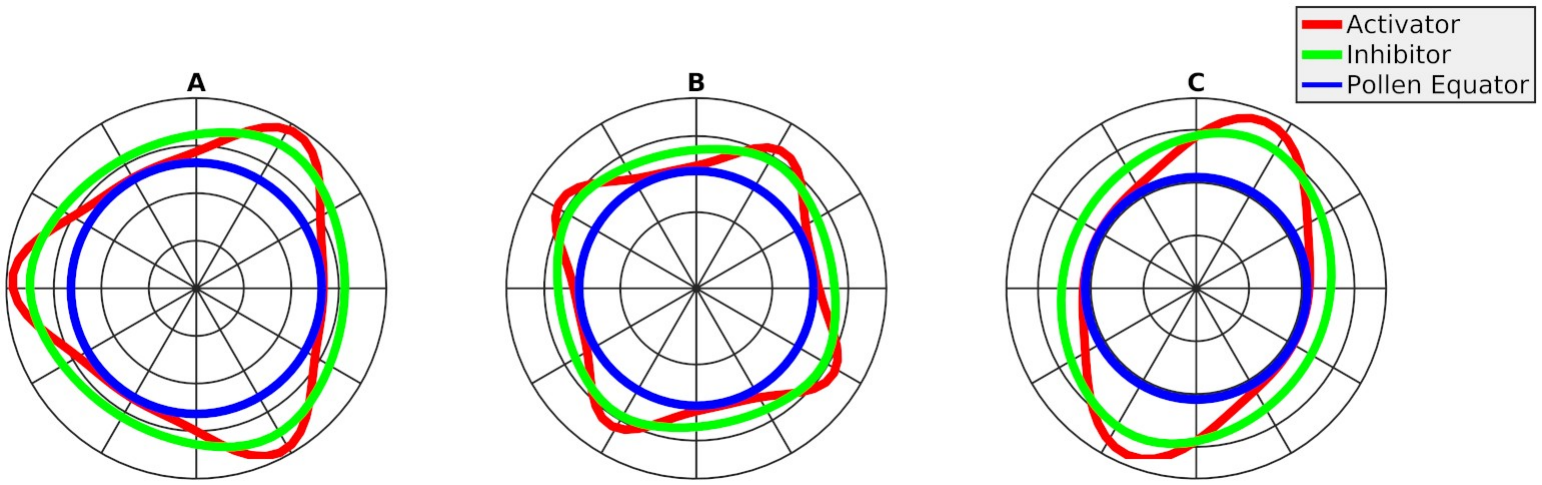
Examples of patterns observed on the 1D domain. The 1D domain representing the pollen equator is shown in blue, the concentration of the activator is in red, and the concentration of the inhibitor is in green. We could commonly observe a three-spike pattern (A), a four-spike pattern (B), and a two-spike pattern (C). The concentration of the activator fluctuates between zero and its maximum, while the concentration of the inhibitor never gets reduced to zero, even between the spikes. The results were obtained running Eqs 1 and 2 with parameter values given in Table 1.

We implemented the coupled GM equations in FlexPDE, a finite-element partial differential equation (PDE) solving environment, as described in the Materials and Methods. To reproduce the aperture patterning conditions of Arabidopsis, a domain was modeled with a size that corresponded to the size of wild-type haploid (1n) pollen grains with an observed front-view surface area averaging 550 *μm*^2^ [13], hereafter referred to as the WT domain size. We used two distinct types of domains in the model: a one-dimensional (1D) domain representing the equator of the pollen grain and a three-dimensional (3D) domain representing the surface on the pollen grain. We explored parameter values that determine the model’s kinetics on the 1D WT domain to find values that would produce three equally spaced spikes, the areas with increased morphogen concentration, to match the aperture patterns of wild-type Arabidopsis pollen grains (Fig 3A; Table 1).

**Table 1:** Parameter values used in the model to reproduce wild-type patterning.

Parameter	Value	Description
D_A_	1.1 *μ*m^2^ s^−1^	diffusion of activator
D_H_	54 *μ*m^2^ s^−1^	diffusion of inhibitor
*μ*_*A*_	0.21 s^−1^	decay of activator
*μ*_*H*_	0.6 s^−1^	decay of inhibitor
*ρ*_1_	0.2 s^−1^	rate of morphogen reaction
*ρ*_2_	0.2 *μ*M^−1^ s^−1^	rate of morphogen reaction
*ρ*_*A*_	0.003 *μ*M	basal growth of activator
*ρ*_*H*_	0.0001 *μ*Ms^−1^	basal growth of inhibitor

We then verified that these parameter values satisfy the conditions for Turing-type patterning [32]. The chosen parameter values were determined to be within the Turing pattern-forming regime. The molecular mechanisms responsible for regulation of the number of apertures and their positioning are still essentially unknown, thus precluding estimations of kinetics for the molecules involved. Therefore, to determine how much change our model could possibly tolerate while still producing Turing patterns, we tested a range of parameter values. We found that when any single parameter was varied between 40% and 270% from the value in Table 1, the criteria for the formation of Turing patterns [32] remained satisfied, indicating that the model is quite robust in its ability to generate patterns.

To verify that our model can reliably reproduce the patterning of Arabidopsis pollen grains, we then increased the domain to match the larger size of the diploid (2n) Arabidopsis pollen grains (hereafter referred to as the larger-size domain). 2n Arabidopsis pollen grains have an average front-view surface area of 750 *μ*m^2^ and often develop patterns with four equatorial apertures [13]. With the base parameters from Table 1, our model produced mostly four-spike patterns on the larger-size domain (Fig 3B), similar to patterns seen in Arabidopsis mutants with 2n pollen. To test if a change of the same magnitude in the opposite direction would also change the number of spikes produced by our model, the domain size was decreased to correspond to a front-view surface area of 350 *μ*m^2^. In this smaller-size domain the number of spikes was reduced to two (Fig 3C).

To test how our model behaved in 3D, we started out with the same parameter values as in 1D. Running initial simulations on the 3D domain, we observed that the WT domain produced mostly three-spike patterns, while the larger-size domain produced mostly four-spike patterns, consistent with the results of the 1D model. The three-spike patterns generated in both 1D and 3D had spikes equally spaced around the equator. However, the four-spikes generated by the 3D model were located at the corners of a tetrahedron. After these initial verifications, we used both the WT domain and the larger-size domain to run our simulations. These two domain sizes, corresponding, respectively, to the sizes of 1n and 2n Arabidopsis pollen grains, were thus capable of reproducing aperture patterns observed *in vivo*.

### Spike number changes in response to domain size

**Fig 4:**
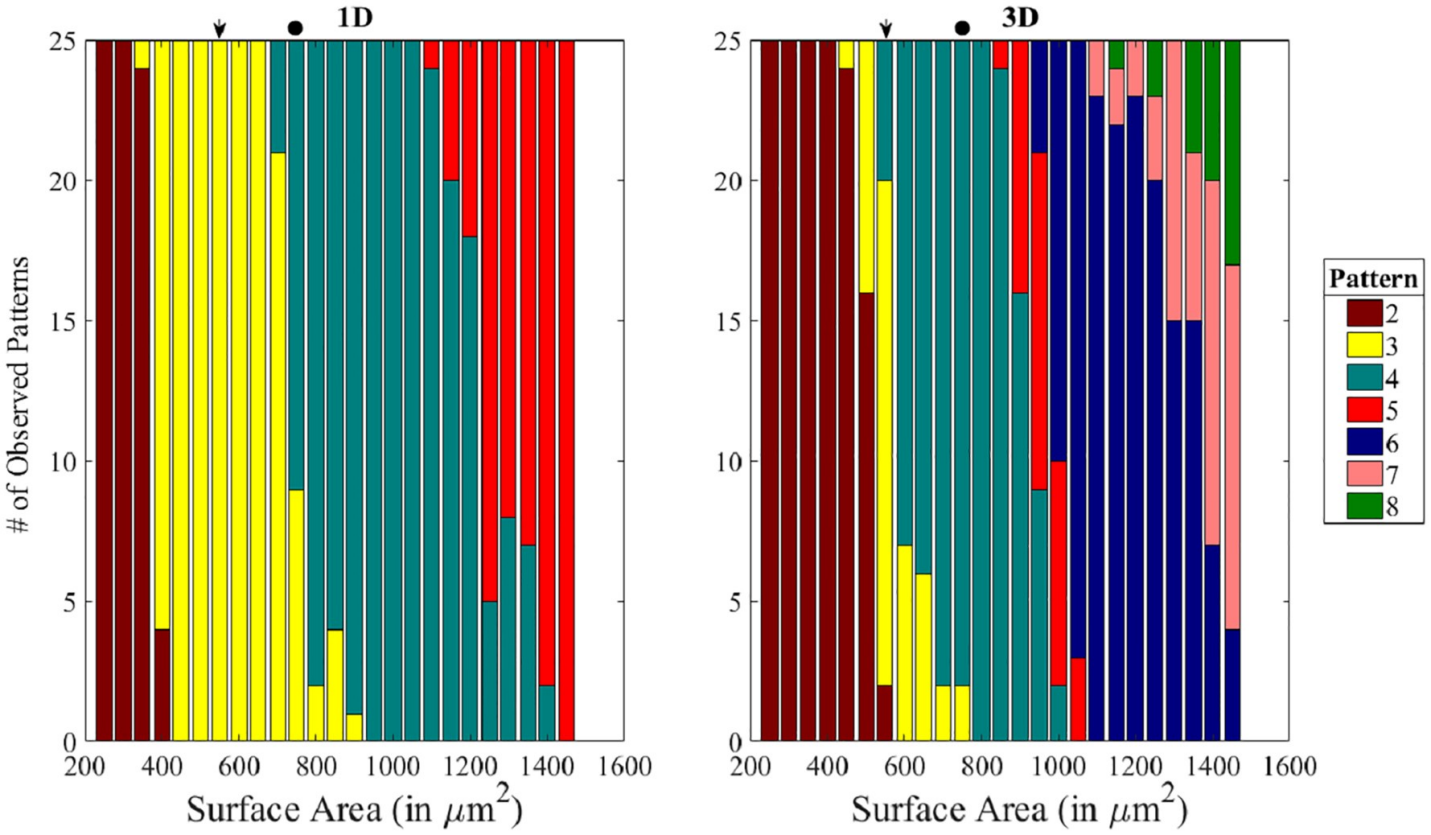
Increase in the domain size can lead to an increase in spike number. The distribution of patterns produced using domains corresponding to surface areas ranging from 250 *μ*m^2^ to 1450 *μ*m^2^ in 1D (left) and 3D (right). As the surface area increased, patterns with more spikes were produced. Arrows mark the wild-type domain and circles mark the larger-sized domain. In this and all subsequent figures, unless stated otherwise, the results were obtained by running Eqs 1 and 2 with parameter values given in Table 1.

Based on the observations that different numbers of spikes can be produced strictly in response to changes in domain size, we decided to systematically determine the effects of domain size on the produced patterns. To accomplish this, we varied the domain size while keeping all of the model parameters at their original values (Table 1). We performed this domain-size analysis for both the 1D and 3D domains and cataloged the resulting patterns. For both domain types, increasing the domain size resulted in a higher number of spikes (Fig 4). The three-spike pattern associated with the wild-type 1n pollen was the most common pattern for 1D domains corresponding to a range of areas between 400 *μ*m^2^ and 700 *μ*m^2^ (95% of simulations, n = 175). The 3D domain, however, was more sensitive to deviations from the original size of the WT domain: the three-spike pattern was predominant (72%, n = 25) only for the area of 550 *μ*m^2^, corresponding to the wild-type domain. The 1D domain produced patterns ranging from two to five spikes, while the 3D domain was capable of producing patterns with up to eight spikes. These results suggest that the domain size, in the absence of any other kinetic or geometric changes, could be responsible for differences in aperture number and patterning.

### The number of spikes is affected by the model kinetics

Higher-ploidy pollen grains have larger size, but they may also have other unknown changes responsible for the changes in aperture patterning. After observing that domain size affects the number of spikes produced, we tested what other mechanisms could be responsible for the aperture phenotypes associated with higher-ploidy pollen. To accomplish this, we performed a one-parameter-at-a-time analysis [33], varying the model parameters in both the WT and the larger-size domains. Parameter values from 20% to 300% of their original values (Table 1) were tested in increments of 20% and the resulting numbers of spikes were tabulated.

#### Increased morphogen diffusion reduces spike number

**Fig 5:**
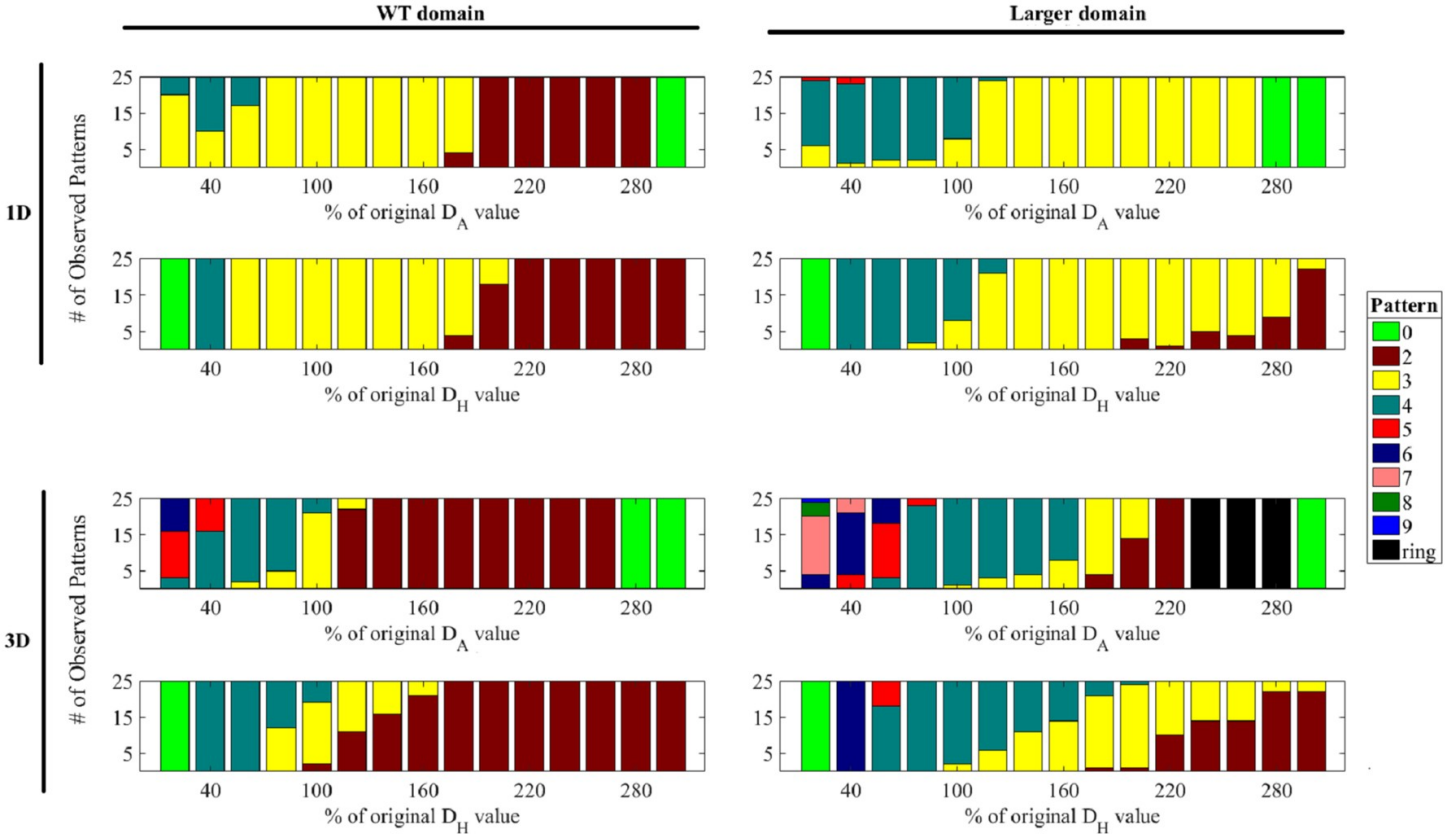
Increase in the diffusion rate leads to a decrease in spike number. In all domains, an increase in diffusion of either the activator (*D*_*A*_, top two rows) or the inhibitor (*D*_*H*_, bottom two rows) leads to a decrease in the number of spikes. The ring-like pattern had increased morphogen concentration along a great circle. The values of the diffusion parameters were varied from 20% to 300% of their original values from Table 1.

Diffusion determines how far and how fast the morphogens disperse through their environments before they are removed or decay. We observed that varying the diffusion rates of the two morphogens changed the number of spikes our model was able to produce (Fig 5). Both WT and larger-sized domains in 1D (Fig 5, Top) and 3D (Fig 5, Bottom) showed the trend that an increase in diffusion led to a decrease in spikes. In addition, more complicated patterns were observed in the larger-size domain in 3D (discussed further below). Together, these data indicate that an increase in one of the diffusion terms would decrease the number of spikes produced.

#### Increased morphogen decay increases spike number

**Fig 6:**
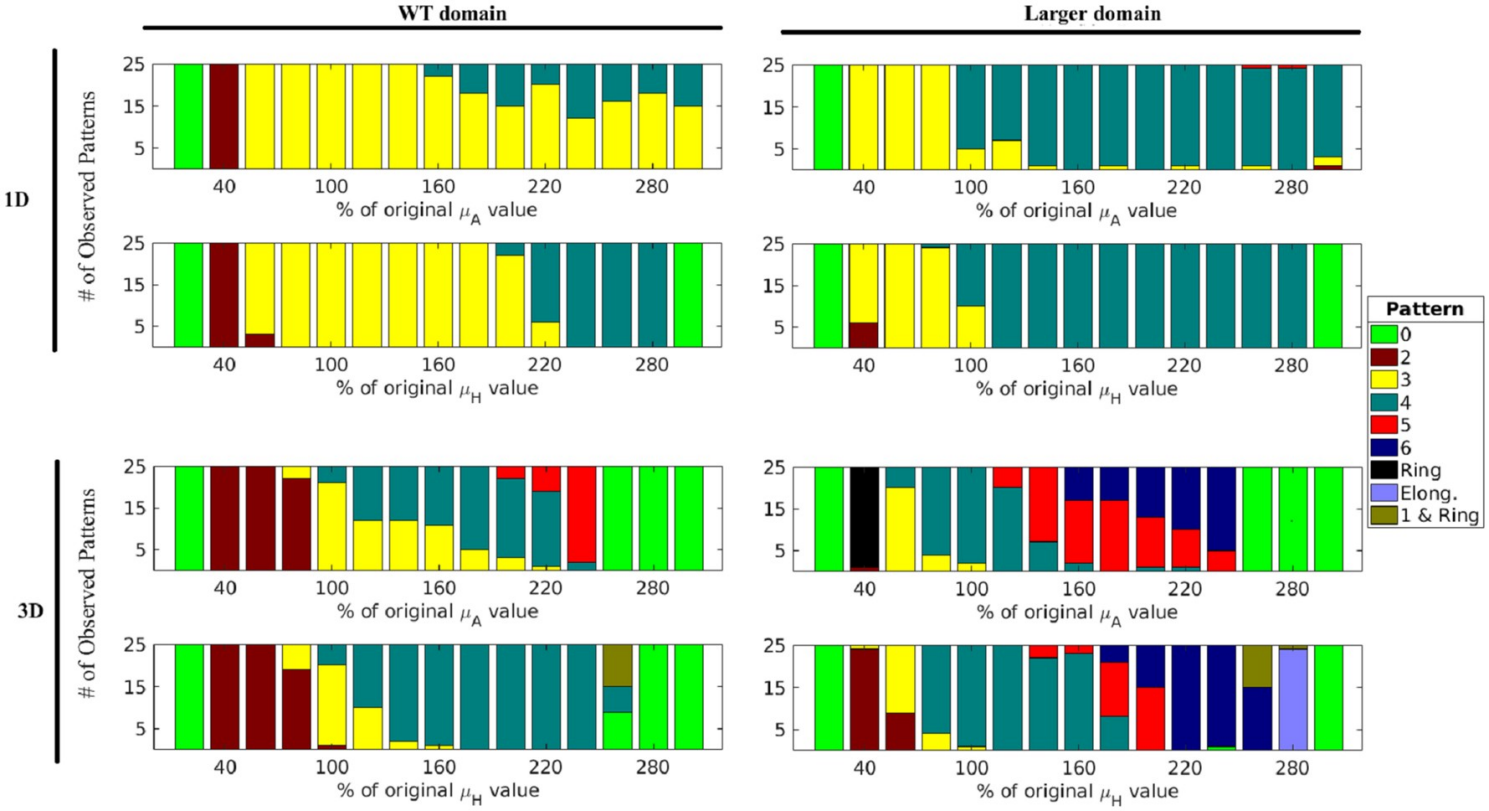
Increase in the morphogen decay leads to an increase in spike number. In both domains, an increase in decay of either the activator (*μ*_*A*_, top rows) or the inhibitor (*μ*_*H*_, bottom rows) led to an increase in the number of spikes. “Ring” represents increased morphogen concentration along a great circle, “1 and Ring” represents a single spike located at a pole of the sphere combined with a ring located on the opposite side of the sphere, and “Elong.” represents six elongated spikes located on the edges of a tetrahedron. The values of the decay parameters were varied from 20% to 300% of their original values from Table 1.

The decay rate determines how fast the morphogens decay or are otherwise removed from their environment. Changes in this parameter can affect how far the morphogens are able to travel, but not how fast they travel. We found that varying the decay rates of the two morphogens changed the number of spikes produced. With both sizes of the domain and in both 1D (Fig 6, Top) and 3D (Fig 6, Bottom), the observed trend was that an increase in decay of either morphogen increased the number of spikes produced when the tested parameters remained within the pattern-forming regime. In 3D, in addition to the usual spikes, some more complicated patterns, discussed below, were observed.

#### Changes in other parameters do not affect spike patterning

The analysis on the reaction efficiencies, *ρ*_1_ = *ρ*_2_, hereafter referred to as *ρ* (S1 Fig), and the basal growth terms, *ρ*_*A*_ and *ρ*_*H*_ (S2 Fig), had no observable effect on spike number or position, even when varied over a large range.

### Novel patterns observed in the 3D model

**Fig 7:**
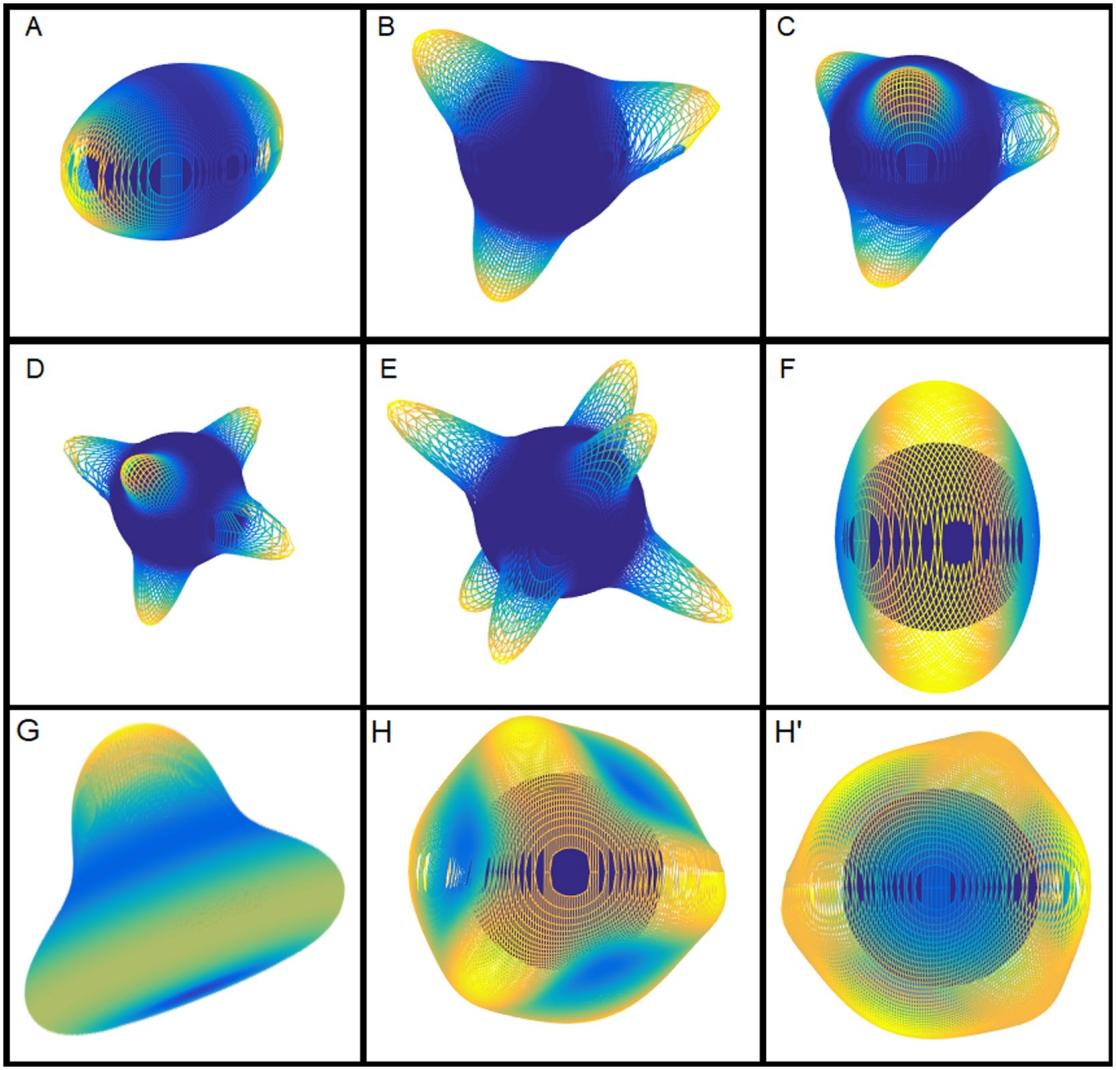
Examples of the different patterns the model simulated on a sphere was able to produce when varying the kinetics. In 3D, the following patterns were commonly observed: (A) two spikes on opposite poles, (B) three spikes equally spaced along a great circle, (C) four spikes located on the corners of a tetrahedron, (D) five spikes, (E) six spikes, with four spikes equally spaced along a great circle and the remaining two spikes on the poles, (F) a ring-like pattern with increased morphogen concentration along a great circle, (G) a single spike and a ring-like pattern, and (H,H’) six elongated spikes located on the edges of a tetrahedron. In the 3D plots, the activator concentration is represented by the height from the initial surface of the sphere (dark blue), color-coded from blue (almost no activator) to yellow (the highest amount of activator).

In 3D, our model tended to produce patterns with various numbers of spikes that were separated from each other by an area where the concentration of the activator was reduced to almost zero (Fig 7A–7E). In addition, in our kinetic experiments we discovered patterns in morphogen concentration that were composed not just of the typical spikes. One of these patterns was the ring-like pattern that had increased morphogen concentration all along a great circle of the spherical domain (Fig 7F). This pattern was observed on the larger 3D domain, in the cases when the diffusion of the activator was greatly increased (Fig 5) or the decay of the activator was sufficiently reduced (Fig 6). Upon further investigation, we determined that the ring-like aperture was created by merging two separate spikes that were initially located on opposite poles (S1 Video). The second type of unusual pattern consisted of a single spike located at a pole of the sphere, combined with a ring located on the opposite side of the sphere (Fig 7G). Additionally, we observed a pattern composed of six elongated spikes located at the edges of a tetrahedron (Fig 7H and 7H’). All of these more complex patterns were formed when the parameter values for the morphogen diffusion or decay were close to the edge of the pattern-forming regime (Figs 5 and 6).

### New aperture mutants with novel phenotypes matching the model predictions were identified in an Arabidopsis genetic screen

To determine if patterns predicted in response to changes in model parameters could be observed *in vivo* in response to genetic perturbations, we performed a forward genetic screen on an ethyl methanesulfonate (EMS)-mutagenized population of Arabidopsis plants. We found mutants belonging to two complementation groups, *macaron* (*mcr*) and *doughnut* (*dnt*), which had pollen aperture phenotypes that have not been previously observed in Arabidopsis, but were predicted by our mathematical model to result from changes in microspore geometry or morphogen kinetics.

**Fig 8:**
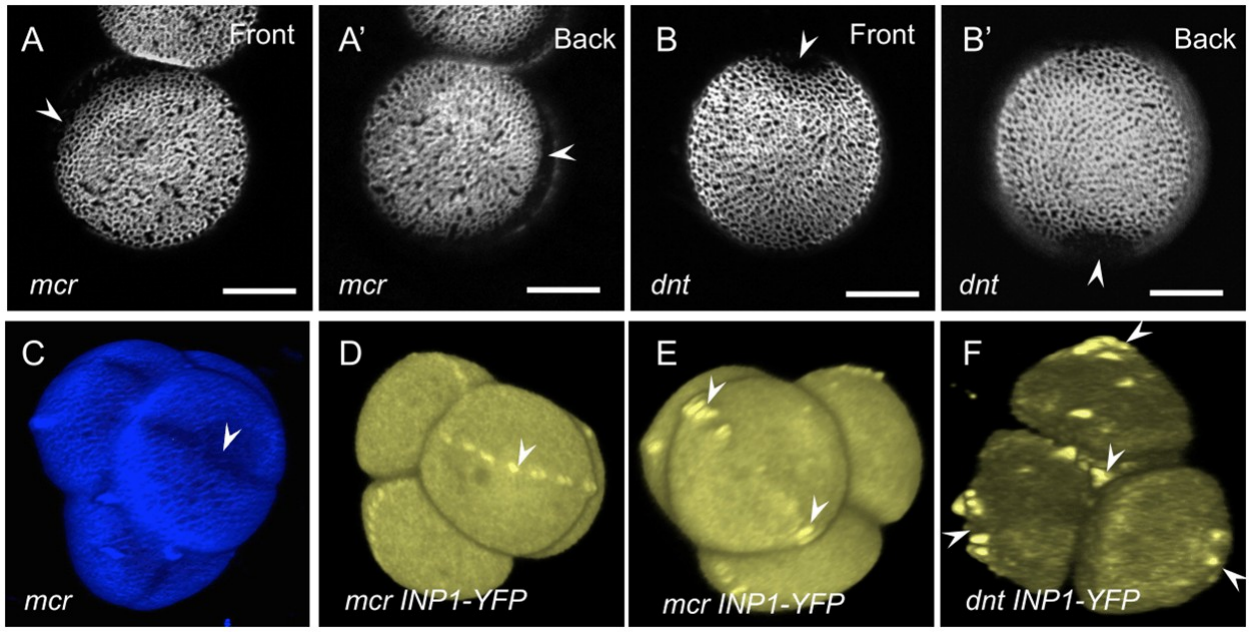
Pollen aperture phenotypes and INP1-YFP localization in the *mcr* and *dnt* mutants. (A-A’) *mcr* pollen has a single ring-like aperture (arrowheads) that runs around the pollen circumference (front and back views of the same pollen grain are shown). See also S2 Video. (B-B’) *dnt* pollen usually has two round apertures (arrowheads) located at diametrically opposite positions (front and back views of the same pollen grain are shown). See also S3 Video. Scale bars = 10 *μ*m. (C) A 3D reconstruction of a tetrad of *mcr* microspores with early exine (blue) and single ring-like apertures (arrowhead) visible. Each aperture runs through the poles of microspores. (D-F) INP1-YFP localization changes in the *mcr* and *dnt* mutants. (D) INP1-YFP forms a single punctate line in *mcr* microspores, passing through the microspore poles and underlying the position of the future single ring-like aperture. See also S4 Video. (E) INP1-YFP localization to two sites close to each microspore equator indicates that the round apertures in *mcr* start their development as two apertures that eventually fuse at the poles. See also S5 Video. (F) In the *dnt* mutant tetrads, INP1-YFP forms punctate circles at the proximal and distal poles of each microspore. See also S6 Video.

Instead of three apertures characteristic of the wild-type Arabidopsis pollen, pollen in the *mcr* mutants developed a single circular aperture that, like a belt, surrounded each pollen grain (Fig 8A and 8A’, S2 Video). To establish positional orientation of the circular aperture in relation to pollen poles and equator, we imaged tetrads of microspores with early signs of apertures and found that this aperture passed through the poles in each microspore (Fig 8C), indicating that the normal longitudinal orientation of apertures was not disrupted in the *mcr* mutant.

By crossing the *DMC1pr:INP1-YFP* reporter construct [14] into the *mcr* background, we determined that in this mutant the aperture factor INP1 assembled in a single circular line (Fig 8D, S3 Video). Moreover, through this imaging approach we established that the single aperture in the mature *mcr* pollen in fact originates as two apertures that form at the opposite sites close to the equator of each microspore, and then become connected at the poles (Fig 8E, S4 Video). Thus, the *mcr* mutation affected the number, but not the furrow-like morphology or the equidistant longitudinal distribution of apertures. The patterning behavior of these *mcr* mutant pollen matches the ring-like apertures that were observed in the mathematical model when the decay of the activator was low or the diffusion of the activator was high (Fig 7F, S1 Video). It could also potentially be considered similar to the two-aperture pattern since the ring aperture in the *mcr* mutants starts as two separate apertures.

Mutants belonging to the *dnt* complementation group also exhibited a novel aperture phenotype: instead of three furrow-like apertures, pollen developed two round, hole-like, apertures that were significantly wider and shorter than the wild-type apertures, had internal deposits of the exine material sporopollenin, and were located at the opposite sites on the pollen surface (Fig 8B and 8B’, S5 Video). By crossing *DMC1pr:INP1-YFP* into the *dnt* background, we established that, as with *mcr*, in this mutant INP1 became localized to the positions of new apertures. These apertures, however, no longer formed at the pollen equator but rather at its poles (Fig 8F, S6 Video; see S3 Fig for additional examples of *dnt* phenotypes). Therefore, *dnt* mutations simultaneously affected multiple aspects of aperture patterns—morphology, number, and position. The patterning of the *dnt* mutants qualitatively resembles the two-spike patterns observed in the model (Fig 7A).

### Size, ploidy, and microsporogenesis of the *mcr* and *dnt* pollen are not affected

Our previous findings that pollen ploidy and/or size have a strong influence on aperture number (Fig 1; [13]) prompted us to pay attention to these parameters in *mcr* and *dnt*. The size of both *mcr* and *dnt* pollen did not differ from the size of the normal 1n pollen produced by the 2n wild-type Arabidopsis (S4 Fig). In addition, crosses of *mcr* and *dnt* with the diploid Landsberg and Columbia accessions, as well as with some other diploid Arabidopsis strains, resulted in fully fertile plants that produced homogeneous pollen and themselves generated normal progeny, without any defects indicative of abnormal ploidy. Similarly, the results of ploidy manipulations that were later performed on *mcr* and *dnt* (see below) were also consistent with the original mutants behaving like the 2n plants and producing 1n pollen. We conclude, therefore, that the changes in aperture number in these mutants were not caused by a size- or ploidy-sensitive mechanism. Also, in both *mcr* and *dnt* mutants, pollen developed through a normal tetrahedral tetrad stage (Fig 8C–8F, S3, S4 and S6 Videos), consistent with the normal progression of meiosis and cytokinesis during microsporogenesis.

### Increase in ploidy/size has different effects on apertures in the *mcr* and *dnt* pollen

Our mathematical model predicted that kinetic changes, such as increased rates of diffusion of an activator (*D*_*A*_) or an inhibitor (*D*_*H*_), can result in the formation of wild type-sized pollen with two apertures (Fig 5). The model further predicted that, if similar changes in diffusion occurred on a larger domain (equivalent to changes in size from 1n to 2n pollen), the number of apertures should correspondingly change from four, typically produced by the 2n/larger-size pollen, to three.

Because both *mcr* and *dnt* pollen grains, at least initially, develop two apertures, we decided to use them to test the model’s predictions by creating *mcr* and *dnt* plants that would produce larger (2n) pollen and assessing the number of apertures in these pollen grains. By treating *mcr* plants with colchicine, as we did previously for wild type [13], we generated tetraploid (4n) *mcr* plants that produced 2n pollen through a tetrad stage. Additionally, both *mcr* and *dnt* mutants were crossed with the *osd1* mutation, which results in the omission of the second meiotic division and leads to the formation of 2n pollen through a dyad stage [13, 34] (Fig 1G). In all cases, the size of the resulting 2n *mcr* and *dnt* pollen was similar to the size of other 2n pollen (S4 Fig), as well as to the size of the larger domain that was used in our simulations.

**Fig 9:**
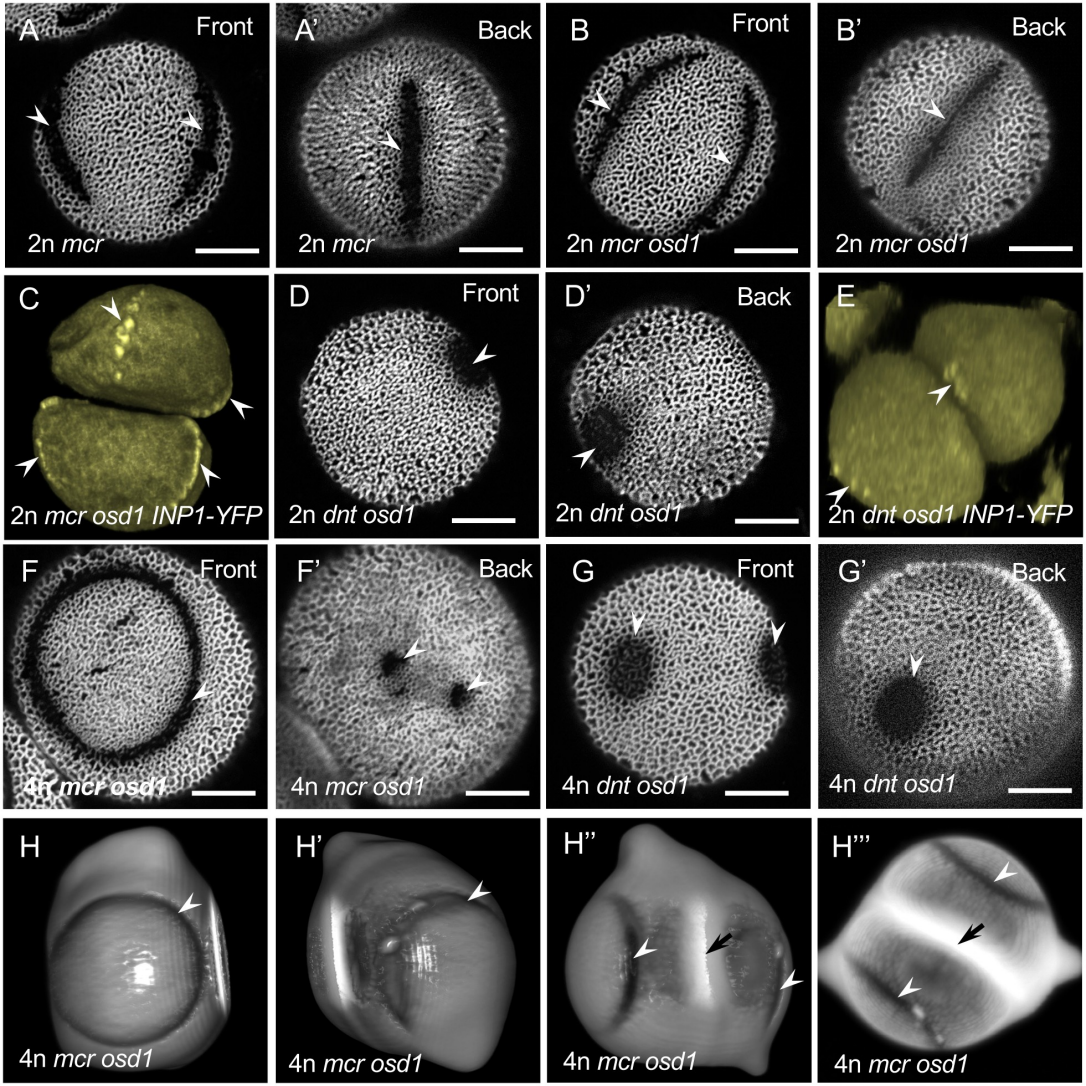
Effects of changes in pollen ploidy/size on pollen aperture patterns in the *mcr* and *dnt* mutants. (A-B’) Diploid *mcr* pollen forms three apertures (arrowheads), independently of the meiotic product arrangement. (A-A’) Front and back views of the 2n *mcr* pollen produced by a 4n plant through a tetrad formation. (B-B’) Front and back views of the 2n *mcr* *osd1* pollen produced through a dyad formation. (C-C’) Diploid pollen of *dnt* *osd1*, similar to the haploid *dnt* pollen, usually has two round apertures (arrowheads). See also S7 Video. (D) INP1-YFP forms three longitudinal lines (arrowheads) in diploid *mcr* *osd1* dyads. A 3D reconstruction of a *mcr* *osd1* *INP1-YFP* dyad is shown. See also S8 Video. (E) INP1-YFP forms puncta at the proximal and distal poles of microspores in diploid *dnt* *osd1* dyads. A 3D reconstruction of a *dnt* *osd1* *INP1-YFP* dyad is shown. See also S9 Video. (F-F’) Increase of pollen ploidy in *mcr* *osd1* to 4n most commonly leads to the formation of a ring-like aperture on one side of the pollen grain (F, arrowhead) and one or several hole-like apertures (F’, arrowhead) on the other side. (G-G’) Increase of pollen ploidy in *dnt* *osd1* to 4n usually increases the number of round apertures to more than two. (H-H”’) The ring-like apertures (arrowheads) in the 4n mcr osd1 dyads are located on the distal end of each microspore, thus placing the hole-like apertures at the proximal end, near the intersporal callose wall (black arrows). Shown are different views of a 3D reconstruction of a late-stage dyad (H-H”) and a maximum intensity projection of a confocal z-stack of the same dyad (H”’) See also S10 Video. Scale bars = 10 *μ*m.

In wild type, such ploidy-increasing manipulations commonly lead to the development of pollen with four or six apertures (Fig 1B–1D’ and 1G; [13]). In the case of *mcr*, independently of the mechanism through which the 2n pollen was generated and consistent with the model’s predictions, the pollen switched to producing three equidistant apertures (Fig 9A–9B’). To determine positional orientations of these three apertures in the *mcr osd1* pollen, we used INP1-YFP as a marker for aperture positions. We found that, in each microspore, INP1-YFP localized to three equidistant longitudinal lines, which were not aligned between the sister microspores (Fig 9C, S7 Video), unlike in wild-type tetrads where the lines of INP1 align between sister microspores [12–14].

In contrast to the 2n *mcr* pollen, in the case of the 2n *dnt* pollen (*dnt osd1*), pollen grains retained two apertures that were morphologically identical to the apertures of the 1n *dnt* pollen (Fig 9D and 9D’, S8 Video). Also, the INP1-YFP signal was found at the poles of microspores in the diploid dyads (Fig 9E, S9 Video), indicating that, like in their 1n counterparts, the two apertures in the 2n *dnt osd1* pollen developed at the poles.

### Further increase in pollen ploidy affects aperture patterns in both *mcr* and *dnt* mutants

In Arabidopsis, pollen with ploidy higher than 2n commonly develops complex and irregular aperture patterns, often consisting of ring-shaped apertures (Fig 1E–1F’; [13]). To test what effect further ploidy increase will have on the *mcr* and *dnt* pollen, we took advantage of the fact that the *osd1* mutation affects meiosis not only in pollen but also in eggs, thus leading to doubled ploidy after self-pollination [13, 34]. Using this approach, we generated tetraploid (4n) *mcr* *osd1* and *dnt* *osd1* plants that produced 4n pollen through the dyad stage. As with other versions of the *mcr* and *dnt* pollen, which were similar in size to the other pollen of the same ploidy level, the size of the 4n *mcr* and *dnt* pollen did not differ from the size of 4n *osd1* pollen (S4 Fig) or from the other 4n pollen types that we previously studied [13].

In the 4n *mcr* *osd1* pollen, the predominant aperture pattern (81%, n = 85) consisted of a ring-shaped aperture located on one side of the pollen grain and 1-2 small, hole-like apertures on the opposite side of the pollen grain (Fig 9F and 9F’; S5 Fig). The remaining grains had variations of this pattern: for example, a ring-shaped aperture and a furrow (S5C and S5C’ Fig), or two ring-shaped apertures on the opposite sides of a grain (S5D and S5D’ Fig). Interestingly, this unusual aperture pattern strongly resembled the ring-and-spike pattern produced by the model (Figs 6 and 7G). To determine where on the surface of 4n *mcr* *osd1* pollen the ring and the hole-like apertures were located, we analyzed the late-stage dyads in which apertures were already recognizable. We found that the rings were located at the distal end of microspores, thus placing the hole-like apertures at the proximal end, near the intersporal callose wall (Fig 9H–9H”’, S10 Video.

In the 4n *dnt osd1* pollen, the shape and size of apertures was still unchanged compared to the 1n *dnt* or 2n *dnt osd1* (Fig 9G and 9G’); however, their numbers increased. Most of the pollen grains had either three (59%, n = 81) or four (36%, n = 81) hole-like apertures (Fig 9G and 9G’, S3A and S3A’ Fig), with some having up to eight apertures (S3B and S3B’ Fig, S11 Video).

### Transient or continuous external stimuli can specify spike number and position

It is not clear if some kind of pre-patterning information (e.g. related to meiosis or cytokinesis, such as positions of meiotic spindles or last contact points between sister microspores) influences the number and positions of apertures. It is also unknown if pre-patterns might act as transient or sustained stimuli. To test whether pre-patterning can dictate final patterns of spikes, we subjected the model to either a transient stimulus, applied as an initial condition, or a sustained stimulus which remained active through the entire simulation time.

#### Both transient and continuous stimuli can affect spike patterning

**Fig 10:**
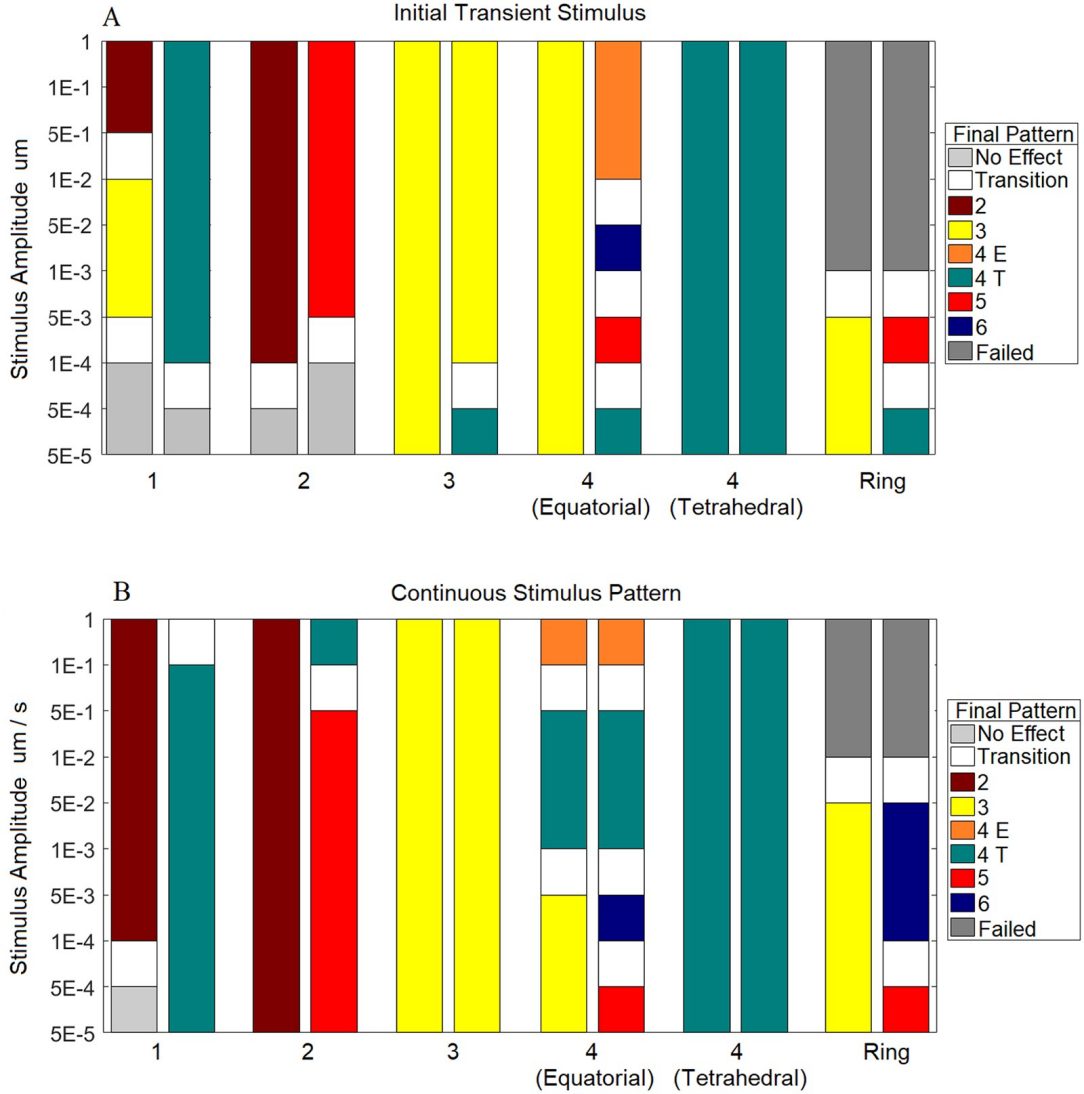
Adding a transient or continuous stimulus to the 3D domain. The type of pattern produced depends on the stimulus pattern, its amplitude, and whether it is transient (A) or continuous (B). Stimuli with larger amplitudes or with three or four spikes were able to better specify the final pattern produced. Left bars show the results for simulations performed on the WT domain; right bars show the results for simulations performed on the larger-size domain. “4 E” represents four spikes on the equator; “4 T” represents four spikes at the corners of a tetrahedron. Some ranges of stimulus amplitudes produced patterns that were not consistently the same; these ranges are marked as “transition”. The ring-patterned transient stimulus caused the PDE solver to fail when the amplitude was too large; these are marked as “Failed”. “No Effect” marks the situations when the produced pattern did not correspond to any positions of the applied stimulus. When a stimulus had an effect on a pattern, we observed at least one of the resulting spikes in all simulations to form at a position where the stimulus was applied. The results in panel A were obtained running Eqs 1 and 2. Panel B was produced using Eqs 2 and 4.

Patterns produced with the 3D geometry can be influenced by an initial transient stimulus added to the system (Fig 10A). A three-spike transient stimulus of all amplitudes in the wild-type domain, and of amplitudes above 10^−4^ *μ*M in the larger-size domain always led to a three-spike pattern. An initial unpatterned stimulus of any amplitude was unable to change the pattern produced. Also, the low-amplitude stimuli consisting of one or two spikes were unable to influence the pattern produced. In these cases, after the transient stimulus disappeared, the resulting pattern had no spatial correlation with this stimulus.

In multiple cases, addition of continuous stimuli influenced the patterns produced by the model in 3D (Fig 10B). In the WT domain, applications of the two-spike and three-spike equatorial stimuli, as well as of the four-spike tetrahedral stimulus, always led to the development of their respective patterns. Similarly, in the larger-size domain, the three-spike equatorial stimulus and the four-spike tetrahedral stimulus always led to the production of their respective patterns. The continuous stimuli were able to specify patterns even when they had a lower amplitude than the corresponding transient stimuli.

## Discussion

In *Arabidopsis thaliana* and in many other eudicot species, aperture patterns on the pollen surface are influenced by mutations that affect size and ploidy of the pollen grains [13, 35–38]. It is, however, unknown if this aberrant patterning is caused by the increase in the pollen grain size or by changes in some other factor(s) linked to ploidy. To explore possible mechanisms responsible for pollen aperture patterning, a mathematical model based on the Gierer-Meinhardt equations was developed. This GM-based model successfully recapitulated the triaperturate equatorial patterning of the wild-type Arabidopsis pollen both in 1D and in 3D geometries.

Our simulations demonstrated that the domain size, as well as morphogen diffusion and decay, could greatly influence the patterns produced by the morphogens. When the kinetic parameters of the morphogens were kept constant, a domain with a larger surface area generated patterns with a larger number of spikes representing apertures: under these conditions more spikes could fit on the domain.

Areas between the morphogen spikes exhibited reduced concentrations of the two morphogens, the activator and the inhibitor. Importantly, in these areas the concentration of the activator went down almost to zero, whereas the concentration of the inhibitor, although decreased, still remained at a relatively high level (Fig 3). The size of these areas of inhibition depends on the kinetics of the system—in particular, on the diffusion and decay of the two morphogens. If the diffusion of a morphogen is increased, the morphogen can travel further through the domain, leading to the decrease in the number of spikes produced. When the morphogen decay rate is decreased, the morphogens are also able to travel further and a similar decrease in the number of resulting spikes was observed. These two types of kinetic changes both extend the morphogen’s range and increase the area of inhibition, thus limiting the number of spikes that can be formed. The opposite changes to the morphogen kinetics—decrease in the diffusion or increase in the decay—will decrease the area of inhibition and correspondingly increase the number of spikes. The other parameters used in the model, *ρ*, *ρ*_*A*_, and *ρ*_*H*_, do not influence the dispersion of the morphogens but instead control the rate of morphogen production at any given location. It appears that the distance the morphogens travel, rather than the amounts of morphogens, can affect the number and patterning of spikes. Formation of the areas of inhibition may be the basis of the mechanism acting in Arabidopsis pollen grains.

To explore how sensitive our model was to the influence of additional stimuli, we first tested transient stimuli, in the form of either patterned or unpatterned initial conditions, followed by continuous stimuli. We found that only a strong transient stimulus could influence the final pattern produced by the model, whereas the weak stimuli, both patterned and unpatterned, were unable to affect the final spike pattern. Similarly, very weak continuous stimuli were often unable to influence patterning. Yet, overall, continuous stimuli of low amplitudes were more successful in specifying patterns than the corresponding transient stimuli. We found that in the presence of either transient or continuous stimuli with three spikes and the amplitude above 10^−4^ *μ*M, the larger-sized 3D domain produced only three-spike solutions instead of the typical four, indicating the strong effect of these types of stimuli on the resulting pattern. This suggests that if there exists some form of *in vivo* pre-patterning stimulus specifying three apertures, then the stimulus is likely to be small since pollen is capable of overcoming its influence and producing a different number of apertures (Fig 1G).

Our model was able to recreate elongated, furrow-like spikes resembling Arabidopsis apertures (Fig 7) only when we used extreme parameter values that were close to the levels at which the model reaches bifurcation and stops producing patterns. This indicates that the elongation is not robust to perturbations to the model, and that the formation of elongated apertures *in vivo* may be caused by other mechanisms that were not captured by our model.

Interestingly, the distribution of four apertures, equally spaced around the equator and commonly observed in the 2n Arabidopsis pollen, was not captured well in the 3D geometry. The majority of the four-spike patterns produced in 3D had the spikes centered on the corners of a tetrahedron (Fig 7C). In contrast, in 1D the model assumes that the process of initial aperture patterning is restricted to the equator of the microspores, and here four-spikes were the predominant pattern for larger domain sizes and for many of the tested parameter values. If the process that initially positions aperture factors is restricted to the pollen equator, then the 1D domain would be a good fit for the patterning expected in Arabidopsis mutants with larger pollen grains.

In addition to the patterns of three or more apertures that were previously observed in Arabidopsis pollen, our model also predicted that under some conditions pollen might be able to produce two apertures or a ring-shaped aperture that could be created by the fusion of two apertures. The aperture pattern of the *mcr* mutant, with a ring-like aperture produced by joining of the two initial apertures (Fig 8E, S4 Video), has a similar phenotype and appears to be generated in the same way as the ring-like patterns in the model (S1 Video). However, in the mathematical model, the ring-like patterns developed only on the larger-size domain and either when the morphogen diffusion was at an extreme value, close to the boundary of the pattern-forming region, or when the morphogen decay was sufficiently reduced. On the WT domain, the ring-like pattern was not formed with any choice of individually changed parameters, nor was it formed in the presence of a stimulus mimicking the ring-like pattern. Therefore, it is possible that in the *mcr* mutants, which have pollen of normal size, the mutation leads to multiple kinetic changes that together contribute to the production of the ring-like aperture. An alternative possibility is that the apertures in the *mcr* mutant correspond to the two-spike pattern, which was frequently produced by our model for normal-size domains (e.g. in the cases of increased diffusion or decreased decay for an activator). This hypothesis is consistent with the observation that apertures in the tetrad-stage *mcr* microspores start their development at two separate positions (Fig 8E, S4 Video) and later coalesce into the ring-like pattern. It is, therefore, conceivable that the *mcr* mutation could affect the kinetics of aperture factors and that separate mechanisms could be responsible for positioning the centers of apertures and for promoting aperture elongation that creates their final, furrow-like, morphology.

When pollen ploidy and size were increased in the *mcr* mutant, the behavior of its aperture patterns followed the model’s predictions. The model suggested that, instead of two apertures, three apertures should form if the domain size increased to that of the 2n pollen. Indeed, the 2n *mcr* pollen developed the triaperturate pattern—independently of the way this pollen was produced, through a tetrad or dyad. In the 4n *mcr* pollen, the most common aperture pattern consisted of a ring-shaped aperture on one side of the pollen grain and one or two hole-like apertures on the opposite side. Interestingly, our model was also able to generate this unusual pattern.

In palynological literature, the *mcr* -like aperture pattern is referred to as bi-syncolpate [6], zonacolpate [39], or ring-like [2]. The examples of pollen with this aperture pattern are relatively infrequent in nature: for instance, only 15 species out of almost 3,000 species represented in the palynological database PalDat exhibit this pattern [2]. Interestingly, in the case of one well-documented example, the genus *Pedicularis* includes multiple species with bi-syncolpate pollen, as well as species that produce triaperturate (tricolpate and tri-syncolpate) pollen grains [2, 40, 41]. This suggests a possibility that the variations in aperture patterns among the *Pedicularis* species could be the result of mutations affecting the homologs of *mcr* or related aperture factors.

The phenotype of the *dnt* mutant, with its two round apertures on the opposite poles of the pollen grain, mimics the two-spike patterns produced by the model. This indicates that the *dnt* mutation may affect the useful lifespan of the morphogens or the way they are transported within the microspores. The *dnt* mutation might increase the area of inhibition in microspores, so that only two spikes in the concentration of aperture factors will form.

If there are indeed kinetic changes in the Arabidopsis mutants, it is unlikely that they only affect one of the parameters in the model. To account for this, a multi-parameter kinetics analysis could be performed to test all possible combinations of parameter values. In addition, our mathematical model only considered a single microspore in the shape of a perfect sphere, whereas *in vivo* apertures are formed on the microspores that are not entirely spherical and are joined into tetrads. Differently shaped microspores could be modeled to determine if the surface morphology plays a role in the number and positions of apertures. The local geometric and biophysical properties, such as membrane curvature and pressure from the callose wall that envelopes tetrads, could potentially affect protein kinetics, movement, or positioning and influence such processes as aperture elongation. The identification of the genes affected in the *mcr* and *dnt* mutants will likely provide important insights into the mechanism of pollen aperture patterning that can be used to further refine the mathematical model.

The choice of the Gierer-Meinhardt model in this study was due to the fact that the patterns observed on pollen grains are equally spaced, similar to the patterns produced by the GM model. The GM model has been successfully used to study similar patterns in other biological systems [27, 28], and we applied that model here to better understand specification of aperture positioning. A similar model has previously been used to simulate pattern forming processes, where the underlying interaction network is unknown, to great effect [25]. As more information is gained about the proteins involved in aperture specification, more detailed and biologically realistic models can be developed to explore aperture formation in more detail. For example, we have recently identified the protein kinase D6PKL3 as an aperture factor [16]. This suggests that mechanisms involving protein phosphorylation and dephosphorylation might have to be taken into account in the future models.

In conclusion, we were able to show computationally that aperture formation in Arabidopsis could be controlled by kinetics of aperture factors and by pollen grain size. Following the model’s predictions, we found Arabidopsis plants with the previously unobserved *mcr* and *dnt* aperture phenotypes. These findings demonstrate that mathematical modeling is able to provide valuable insights even when the mechanisms behind the biological phenomena are unknown or when a direct connection between the biological patterning mechanism(s) and the mathematical patterning is not known to exist. Future work with similar qualitative modeling may help to explain the mechanisms responsible for the great variety of patterns that exist in pollen grains across species.

## Materials and methods

### Mathematical model

In the Gierer-Meinhardt equations [27, 28], a short-range activator ($\text{A}(\vec{x},t)$ and a long-range inhibitor ($\text{H}(\vec{x},t)$ are modeled as a system of partial differential equations. Eqs 1 and 2 describe the changes over time of the activator and the inhibitor, respectively. In the equations, D_A_ and D_H_ are the diffusion constants, *μ*_*A*_ and *μ*_*H*_ are the removal/decay constants, *ρ*_*A*_ and *ρ*_*H*_ represent a constant basal growth of each morphogen, and *ρ*_1_ and *ρ*_2_ are the rates of the interaction between the activator and the inhibitor. The magnitudes of *ρ*_1_ and *ρ*_2_ are the same value and they differ only in their units.

$$\begin{array}{c} \frac{\partial A}{\partial t}=D_{A}\nabla^{2}A-\mu_{A}A+\frac{\rho_{1}{\left(A+\rho_{A}\right)}^{2}}{H} \end{array}$$ (1)

$$\begin{array}{c} \frac{\partial H}{\partial t}=D_{H}\nabla^{2}H-\mu_{H}H+\rho_{2}A^{2}+\rho_{H} \end{array}$$ (2)

Two types of domains were used to explore the patterning of the two morphogens. The first was a one-dimensional domain representing the equator of a microspore. The second was the surface of a three-dimensional sphere, representing the surface of a microspore. For all simulations, the initial conditions of the two morphogens were set to be near their diffusion-free steady-state values. Turing-type patterns require the initial conditions to have a level of random noise for patterns to form [26]. Therefore, we tested random noise levels between 10^−15^ *μM* to 10^2^ *μM*, and found that the model produced identical patterns, indicating that within these limits the noise did not influence the resulting patterns. However, levels of noise below 10^−6^ *μM* and above 10^−2^ *μM* resulted in simulations taking longer to run. Therefore, we added a random value between ±5*10^−4^ *μM* to every point of our initial conditions.

The following assumptions and simplifications were made:

1. A single microspore was modeled, thus ignoring any effects that interactions between sister microspores in a tetrad may have on aperture formation.
2. The microspore was modeled as a perfect sphere, thus ignoring any effects the surface morphology may have on the pattern produced.
3. We assumed that the dynamics of the system do not change over time, and therefore our parameter values stayed constant over a single simulation.
4. Because the size of microspores does not change during the stage when aperture patterns are produced, the size of the domain in our model was kept constant for the duration of simulation.
5. All simulations were run until a steady state was reached.

### *In silico* simulations

Simulations of the model (Eqs 1 and 2) were performed using the software FlexPDE (pdesolutions.com). Time and space steps were chosen to ensure numerical stability. To test for possible causes of the change in pollen aperture number and patterning observed in Arabidopsis mutants, the following *in silico* experiments were run:

#### Domain size analysis

To see if domain size alone could account for changes in the number of apertures, we ran the model with varying domain sizes. In both 1D and 3D simulations, surface areas from 250 *μ*m^2^ to 1450 *μ*m^2^, in increments of 50 *μ*m^2^, were each simulated 25 times and the resulting number of spikes were recorded.

#### Kinetics analysis

To test if changes in the kinetics may affect the number of apertures, a one-parameter-at-a-time analysis was performed. The analysis was performed on both the WT domain (550 *μ*m^2^) and on the larger-size domain (750 *μ*m^2^) representing the mutants with larger pollen. The parameter values given in Table 1 were changed from 20% to 300% of their original values in increments of 20%. 25 simulations were run for each parameter value and the resulting number of spikes was recorded.

#### Addition of transient and continuous stimuli

Microspores could receive some pre-patterning information via meiosis/cytokinesis events or through interaction between sister microspores. To simulate the potential input from this pre-patterning information, we added external stimuli to our model. Two different types of external stimuli were tested, an initial transient stimulus and a continuous stimulus.

The initial transient stimulus in the model was accomplished by adding a patterned initial condition on top of the random noise of the activator. The stimulus was added only to the activator because we wanted the patterning of the stimulus to directly influence the pattern produced by our model. A stimulus could have been added to the inhibitor, but an increase in inhibitor concentration alone would lead to a decrease in activator concentration at that location and therefore produce a pattern unrelated to the stimulus. The stimulus was made to resemble the types of patterns that occur in the model: one, two, three, or four spikes equally spaced around the equator, four spikes placed at the corners of a tetrahedron, and a ring-like pattern with increased concentration around the equator. The initial conditions with addition of the transient stimulus resembled Fig 3 in 1D and 3D or Fig 7(A)–7(F) in 3D. These initial spikes were generated using Eq 3, giving the highest stimulus near the location (*x*_0_, *y*_0_, *z*_0_) that we can place anywhere on our simulated pollen grains. Multiple spikes were simulated by adding additional equations at additional specified locations. For the ring-like stimulus we used a similar equation with only the y-coordinate; this gave the ring-like pattern observed in Fig 7(F).

$$\begin{array}{c} F\left(\vec{x}\right)=e^{-({\left(x-x_{0}\right)}^{2}+{\left(y-y_{0}\right)}^{2}+{\left(z-z_{0}\right)}^{2})} \end{array}$$ (3)

The continuous stimulus, simulating a potential mechanism forcing a specified pattern on the pollen grains, was accomplished by adding a forcing function to Eq 1, resulting in Eq 4. For the reasons explained above for transient stimuli, this forcing function, taking the form of Eq 3, only affected the rate of change of the activator. The same patterns as for the transient stimuli were also tested for the continuous stimuli.

$$\begin{array}{c} \frac{\partial A}{\partial t}=D_{A}\nabla^{2}A-\mu_{A}A+\frac{\rho_{1}{\left(A+\rho_{A}\right)}^{2}}{H}+F\left(x\right) \end{array}$$ (4)

### Plant material and growth conditions

Wild-type (Landsberg *erecta*) and mutant Arabidopsis plants were grown at 20 − 22° C with the 16-hour light: 8-hour dark cycle in growth chambers or in a greenhouse at the Biotechnology facility at OSU. The genetic screen that led to the isolation of the *mcr* and *dnt* mutants was performed similar to the previous screen [42]. In brief, M_2_ plants from eight pools of EMS-treated lines of Landsberg *erecta* background (~10,000 plants) were screened for the presence of morphological abnormalities in their pollen (e.g. in size, shape, light reflection, ease of pollen release from anthers) that were identifiable with standard dissecting stereomicroscopes (Zeiss Stemi-2000C and Nikon SMZ745) at 75-80X magnification. For primary screening, pollen did not undergo any treatment. Particular attention was paid to changes in pollen shape, known to be associated with aperture defects [12, 42]. Pollen of the candidates isolated in the primary screen was stained with auramine O as described [13] and observed for exine and aperture defects with confocal microscopy. Confirmed mutants were then backcrossed at least once. *mcr* and *dnt* mutants were crossed with the previously described *DMC1pr::INP1-YFP* line [14] and with *osd1-2/+* plants that were identified as described [34].

### Colchicine treatment

To create plants of higher ploidy, shoot apical meristems of young plants were treated with colchicine as previously described [13], with minor modifications. Because we found that Landsberg plants were very sensitive to colchicine, the amount of colchicine and the number of treatments were reduced compared to the previous study. A 20 *μ*l drop of 0.25% colchicine; 0.2% Silwet L-77 was applied once onto shoot apices before bolting. Conspicuous, larger-than-normal flowers (often found on thicker-than-normal stems) in colchicine-treated plants were allowed to self-pollinate and their seeds were harvested. These progeny were then analyzed for characteristic increase in the size of plant organs and of pollen, and for stable inheritance of these traits.

### Confocal microscopy

Samples for confocal microscopy were prepared as described [13]. Exine of mature pollen stained with auramine O was excited with a 488-nm laser and the emitted fluorescence was collected at 500-550 nm. Tetrads released from stage-9 anthers [43], were placed into Vectashield anti-fade solution (Vector Labs) supplemented with 0.02% Calcofluor White and imaged on a Nikon A1+ confocal microscope with a 100x oil-immersion objective (NA = 1.4) and 5x confocal zoom. The following settings were used to collect signals of fluorophores: YFP—514-nm excitation/ 522-555 nm emission; Calcofluor White and early exine on tetrad-stage microspores—405-nm excitation/ 424-475 nm emission. Z-stacks of tetrads were obtained with a step size of 500 nm and volume-reconstructed using NIS Elements v.4.20 (Nikon).

## Supporting information

S1 Fig Reaction efficiency *ρ* had little to no effect on the number of spikes produced. The distribution of patterns produced by changing the value of *ρ* from 20% to 200% of the value from Table 1 and simulating each parameter set 25 times.
(TIF)

S2 Fig Basal growth terms *ρ*_*A*_ and *ρ*_*H*_ had little to no effect on the number of spikes produced. The distribution of patterns produced by changing the value of *ρ*_*A*_ and *ρ*_*H*_ from 20% to 200% of the value from Table 1 and simulating each parameter set 25 times. The top row corresponds to the *ρ*_*A*_ term and the bottom row is *ρ*_*H*_.
(TIF)

S3 Fig Additional examples of aperture phenotypes found in 1n *dnt* and 4n *dnt osd1* pollen. (A-B’) Although many of the haploid dnt pollen grains have two round apertures, pollen with elongated apertures (arrowhead in (A)) and apertures composed of two closely positioned holes (arrows in (A’, B)) is also present. (C-D’) Tetraploid dnt *osd1* pollen usually has more than two round apertures. (C, C’) An example of a tetraploid *dnt* *osd1* pollen grain with four round apertures (arrowheads). (D, D’) An example of a tetraploid *dnt* *osd1* pollen grain with eight round apertures (arrowheads). See also S10 Video. Scale bars = 10 *μ*m.
(TIF)

S4 Fig Pollen of *mcr* and *dnt* mutants does not differ in size from the wild-type and *osd1* pollen of the same ploidy. Areas of pollen surface visible in the ‘front view’ images were measured for pollen of the genotypes examined in this study. Data are shown as mean ± SD. Pollen sizes are significantly different between the pollen grains of different ploidy (p-value <0.05, indicated by asterisks) but not between pollen of different genotypes that have the same ploidy.
(TIF)

S5 Fig Additional examples of aperture phenotypes found in 4n *mcr osd1* pollen. (A-D’) 4n *mcr* *osd1* pollen commonly develops a ring-shaped aperture displaced to one side of the grain and one or two dot-like apertures on the opposite side. Scale bars = 10 *μ*m.
(TIF)

S1 Video Ring-like pattern produced by the mathematical model. In 3D, using the larger domain and the decay of the activator set to 40% of the value from Table 1, ring-like patterns are created by the joining of two distinct initial spikes.
(AVI)

S2 Video A *mcr* pollen grain with a ring-shaped aperture. Volume reconstruction of a z-stack of confocal sections. To facilitate visualization, the sections were rainbow-colored depending on their positions—from magenta (front view) to blue (back view).
(MOV)

S3 Video A tetrad of microspores from the *mcr* mutant with the fully formed INP1 lines. Volume reconstruction of a z-stack of confocal sections. Microspores express INP1-YFP (yellow), which forms a single line in each of the microspores.
(MOV)

S4 Video A tetrad of microspores from the mcr mutant with the partially formed INP1 lines. Volume reconstruction of a z-stack of confocal sections. Microspores express INP1-YFP (yellow). Each INP1 line starts assembling at the two opposite sites near the microspore equator and progresses towards the microspore poles.
(MOV)

S5 Video A *dnt* pollen grain with two hole-like apertures at the opposite sides. Volume reconstruction of a z-stack of confocal sections. To facilitate visualization, the sections were rainbow-colored depending on their positions—from magenta (front view) to blue (back view).
(MOV)

S6 Video A tetrad of microspores from the *dnt* mutant with the INP1 puncta localized to the microspore poles. Volume reconstruction of a z-stack of confocal sections. Microspores express INP1-YFP (yellow).
(MOV)

S7 Video A diploid dyad of microspores from the diploid *mcr osd1* mutant. Volume reconstruction of a z-stack of confocal sections. Microspores express INP1-YFP (yellow), which assembles into three lines in each microspore.
(MOV)

S8 Video A diploid *dnt osd1* pollen grain with two hole-like apertures at the opposite sides. Volume reconstruction of a z-stack of confocal sections. To facilitate visualization, the sections were rainbow-colored depending on their positions—from magenta (front view) to blue (back view).
(MOV)

S9 Video A diploid dyad of microspores from the diploid *dnt osd1* mutant. Volume reconstruction of a z-stack of confocal sections. Microspores express INP1-YFP (yellow), which aggregates at the opposite poles in each microspore.
(MOV)

S10 Video The ring-like apertures in the 4n *mcr osd1* dyads are located on the distal end of each microspore. Volume reconstruction of a late-stage dyad of microspores in which early exine and apertures are visible.
(MOV)

S11 Video A tetraploid *dnt osd1* mutant pollen grain with eight hole-like apertures. Volume reconstruction of a z-stack of confocal sections. To facilitate visualization, the sections were rainbow-colored depending on their positions—from magenta (front view) to blue (back view).
(MOV)

## References

[1] Kesseler R, Harley M. Pollen: The hidden sexuality of flowers. 3rd. ed Firefly Books; 2009.

[2] Paldat—a palynological database. [internet] www.paldat.org.

[3] Wang R, Dobritsa AA. Exine and aperture patterns on the pollen surface: Their formation and roles in plant reproduction In Annual Plant Reviews Online, Roberts J. A. (Ed.) 2018: 1–40.

[4] Furness CA, Rudall PJ. Pollen aperture evolution–a crucial factor for eudicot success? Trends Plant Sci. 2004;9: 154–158. 10.1016/j.tplants.2004.01.001 15003239

[5] Wodehouse RP. Pollen grains: Their structure, identification and significance in science and medicine. New York and London: McGraw-Hill; 1935.

[6] Blackmore S, Barnes SH. Harmomegathic mechanisms in pollen grains In Pollen and Spores: Form and Function London: Academic Press; 1986 pp. 137–149.

[7] Heslop-Harrison J. An interpretation of the hydrodynamics of pollen. Am J Bot. 1979;66: 737–743. 10.1002/j.1537-2197.1979.tb06277.x

[8] Katifori E, Alben S, Cerda E, Nelson DR, Dumais J. Foldable structures and the natural design of pollen grains. Proceedings of the National Academy of Sciences. 2010;107: 7635–7639. 10.1073/pnas.0911223107 PMC2867878 20404200

[9] Muller J. Form and function in angiosperm pollen. Ann Mo Bot Gard. 1979;66: 593–632. 10.2307/2398913

[10] Ressayre A, Raquin C, Mignot A, Godelle B, Gouyon PH. Correlated variation in microtubule distribution, callose deposition during male post-meiotic cytokinesis, and pollen aperture number across Nicotiana species (*Solanaceae*). Am J Bot. 2002;89: 393–400. 10.3732/ajb.89.3.393 21665634

[11] Takahashi M. Pattern determination of the exine in *Caesalpina japonica* (*Leguminosae: Caesalpinoideae*). Am J Bot. 1989;76:1615–1626. 10.1002/j.1537-2197.1989.tb15146.x

[12] Dobritsa AA, Coerper D. The novel plant protein INAPERTURATE POLLEN1 marks distinct cellular domains and controls formation of apertures in the Arabidopsis pollen exine. Plant Cell. 2012;24: 4452–4464. 10.1105/tpc.112.101220 23136373 PMC3531845

[13] Reeder SH, Lee BH, Fox R, Dobritsa AA. A ploidy-sensitive mechanism regulates aperture formation on the Arabidopsis pollen surface and guides localization of the aperture factor INP1. PLoS Genet. 2016;12: 1–24. 10.1371/journal.pgen.1006060 PMC4866766 27177036

[14] Dobritsa AA, Kirkpatrick AB, Reeder SH, Li P, Owen HA. Pollen aperture factor INP1 acts late in aperture formation by excluding specific membrane domains from exine deposition. Plant Physiology. 2018;176(1): 326–339. 10.1104/pp.17.00720 28899962 PMC5761771

[15] Li P, Ben-Menni Schuler S., Reeder SH, Wang R, Suarez Santiago V, Dobritsa AA. INP1 involvement in pollen aperture formation is evolutionarily conserved and may require species-specific partners. J Exp Bot. 69: 983–996. 10.1093/jxb/erx407 29190388 PMC5965098

[16] Lee BH, Weber ZT, Zourelidou M, Hofmeister BT, Schmitz RJ, Schwechheimer C, Dobritsa AA. Arabidopsis protein kinase D6PKL3 is involved in the formation of distinct plasma-membrane aperture domains on the pollen surface. Plant Cell. 2018;30:2038–2056. 10.1105/tpc.18.00442 30150313 PMC6181024

[17] Altmann T, Damm B, Frommer WB, Martin T, Morris PC, Schweizer D, Willmitzer L, Schmidt R. Easy determination of ploidy level in Arabidopsis thaliana plants by means of pollen size measurement. Plant Cell Rep. 1994;13: 652–656. 10.1007/BF00232939 24196247

[18] De Storme N, Zamariola L, Mau M, Sharbel TF, Geelen D. Volume-based pollen size analysis: an advanced method to assess somatic and gametophytic ploidy in flowering plants. Plant Reprod. 2013;26: 65–81. 10.1007/s00497-012-0209-0 23686220

[19] Matamoro-Vidal A, Prieu C, Furness CA, Albert B, Gouyon PH. Evolutionary stasis in pollen morphogenesis due to natural selection. New Phytol. 2016;209: 376–394. 10.1111/nph.13578 26248868

[20] Heslop-Harrison J. Wall pattern formation in angiosperm microsporogenesis. Symp Soc Exp Biol. 1971;25: 277–300. 4940549

[21] Dover GA. The organization and polarity of pollen mother cells of Triticum aestivum. J Cell Sci. 1972;11: 699–711. 4648502 10.1242/jcs.11.3.699

[22] Sheldon JM, Dickinson HG. Determination of patterning in the pollen wall of Lilium henryi. J Cell Sci. 1998;193: 321–334. 10.1242/jcs.63.1.191 6313711

[23] Sheldon JM, Dickinson HG. Pollen wall formation in Lilium: The effect of chaotropic agents, and the organisation of the microtubular cytoskeleton during pattern development. Planta. 1986;168: 11–23. 10.1007/BF00407003 24233729

[24] Ressayre A, Godelle B, Mignot A, Gouyon PH. A Morphogenetic Model for Pollen Aperture Pattern in Flowering Plants. J Theo Biol. 1998;193: 321–334. 10.1006/jtbi.1998.0704 9735262

[25] Brooks HA, Bressloff PC. Turing mechanism for homeostatic control of synaptic density during *C. elegans* growth Phys Rev E. 2017; 96: 012413 10.1103/PhysRevE.96.012413 29347189

[26] Turing A. The chemical basis of morphogenesis Phil Trans R Soc Lond B. 1952;237: 37–72. 10.1098/rstb.1952.0012

[27] Gierer A, Meinhardt H. A Theory of Biological Pattern Formation. Kybernetick. 1972;12(1): 30–39. 10.1007/BF00289234 4663624

[28] Meinhardt H. Complex pattern formation by a self-destabilization of established patterns: chemotactic orientation and phyllotaxis as examples. C R Biol. 2003;326: 223–237. 10.1016/S1631-0691(03)00018-0 12754941

[29] Barrio RA, Varea C, Aragon JL et al A two-dimensional numerical study of spatial pattern formation in interacting Turing systems. Bull Math Biol. 1999; 61:483–505. 10.1006/bulm.1998.0093 17883228

[30] MacWilliams HK. Numerical simulations of hydra head regeneration using proportion-regulating version of the Gierer-Meinhardt model. J Theor Biol. 1982;99: 681–703. 10.1016/0022-5193(82)90194-1 7183858

[31] Varea C, Aragon JL, Barrio RA. Turing patterns on a sphere. Phys Rev E. 1999;60: 4588–4592. 10.1103/PhysRevE.60.4588 11970318

[32] Edelstein-Keshet L. Mathematical Models in biology Society for industrial and Applied Mathematics, 2005.

[33] Ingalls B. Sensitivity analysis: from model parameters to system behaviour Essays Biochem. 2008;45:177–193. 10.1042/BSE0450177 18793132

[34] d’Erfurth I, Jolivet S, Froger N, Catrice O, Novatchkova M, Mercier R. Turning Meiosis into Mitosis. PLoS Biol. 2009;7(6):e1000124 10.1371/journal.pbio.1000124 19513101 PMC2685454

[35] Tammes PML. On the origin of number and arrangement of the places of exit on the surface of pollen-grains. Groningen: J.H. De Bussy, 1930 82 p.

[36] Ockendon DJ. Cytology and pollen morphology of natural and artificial tetraploids in the Linum perenne group. New Phytol. 1971;70: 599–605. 10.1111/j.1469-8137.1971.tb02561.x

[37] Schifino MT, Moraes Fernandes MI. Induction of polyploidy and cytological characterization of autotetraploids of *Trifolium riograndense* Burkart (*Leguminosae*). Euphytica. 1987;36: 863–872. 10.1007/BF00051871

[38] Hecht A. Colchicine-induced tetraploidy in Oenothera. Proc Indiana Acad Sci. 1941;51: 87–93.

[39] Friis EM, Crane PR, Pedersen KR. Early flowers and angiosperm evolution. Cambridge Univ. Press 2011.

[40] Wang H, Mill RR, Blackmore S. Pollen morphology and infra-generic evolutionary relationships in some Chinese species of *Pedicularis* (Scrophulariaceae). Plant Syst Evol. 2003; 237: 1–17. 10.1007/s00606-002-0188-y

[41] Wang H, Yu WB, Chen JQ, Blackmore S. Pollen morphology in relation to floral types and pollination syndromes in *Pedicularis* (Orobanchaceae). Plant Syst Evol. 2009; 277: 153–162. 10.1007/s00606-008-0112-1

[42] Dobritsa AA, Geanconteri A, Shrestha J, Carlson A, Kooyers N, Coerper D, Urbanczyk-Wochniak E, Bench BJ, Sumner LW, Swanson R, Preuss D. A large-scale genetic screen in Arabidopsis to identify genes involved in pollen exine production. Plant Physiology. 2011;157: 947–970. 10.1104/pp.111.179523 21849515 PMC3192556

[43] Smyth DR, Bowman JL, Meyerowitz EM. Early flower development in Arabidopsis. Plant Cell. 1990; 2: 755–767. 10.1105/tpc.2.8.755 2152125 PMC159928

