# Supplementary figures and images for: Changes in morphogen kinetics and pollen grain size are potential mechanisms of aberrant pollen aperture patterning in previously observed and novel mutants of Arabidopsis thaliana
Source: PLoS Comput Biol. 2019 Feb 28;15(2):e1006800. doi: 10.1371/journal.pcbi.1006800 (PMC6394904; doi:10.1371/journal.pcbi.1006800)

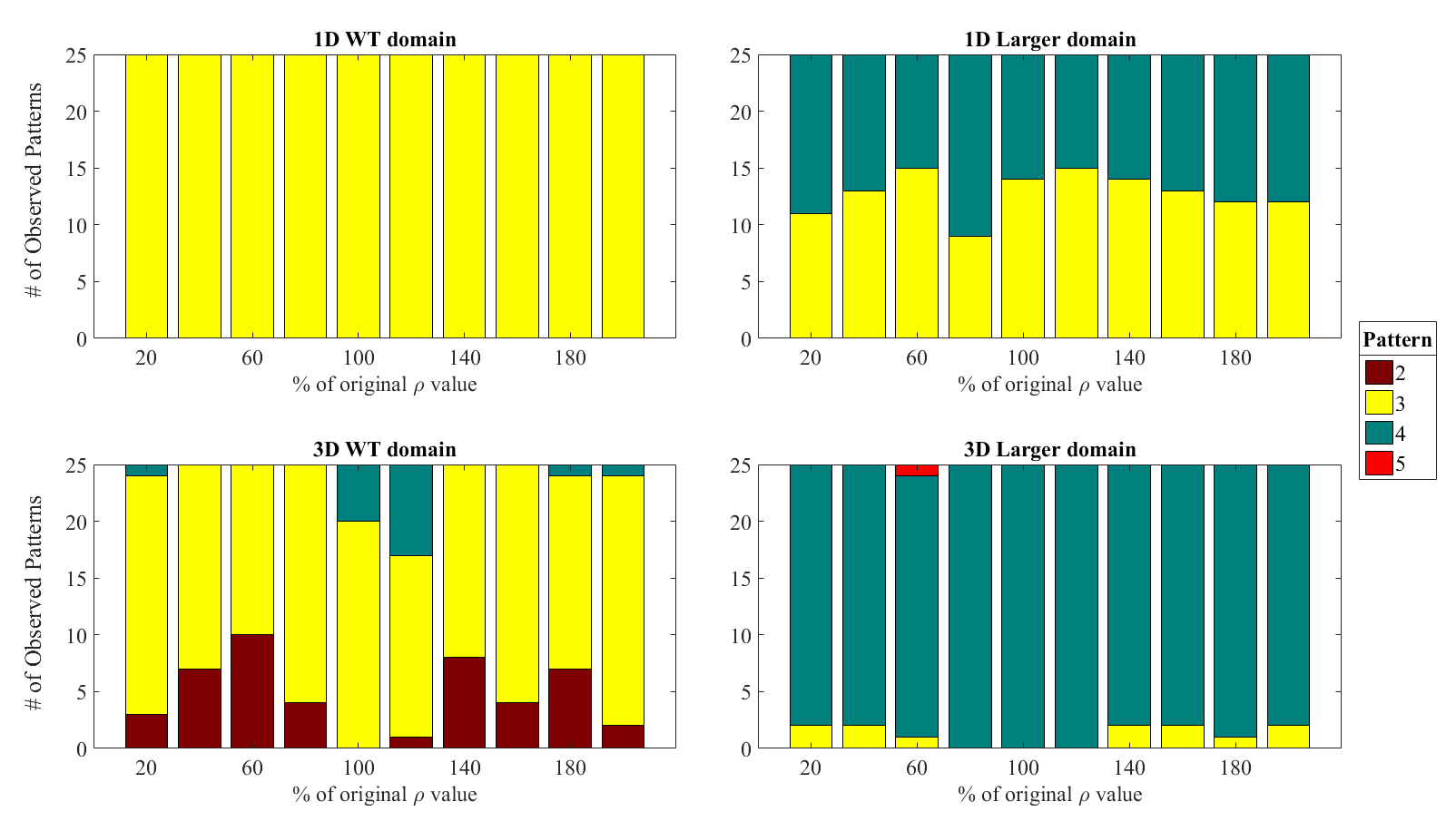

Supplement: S1 Fig — The distribution of patterns produced by changing the value of ρ from 20% to 200% of the value from Table 1 and simulating each parameter set 25 times. (TIF) [file pcbi.1006800.s001.tif]

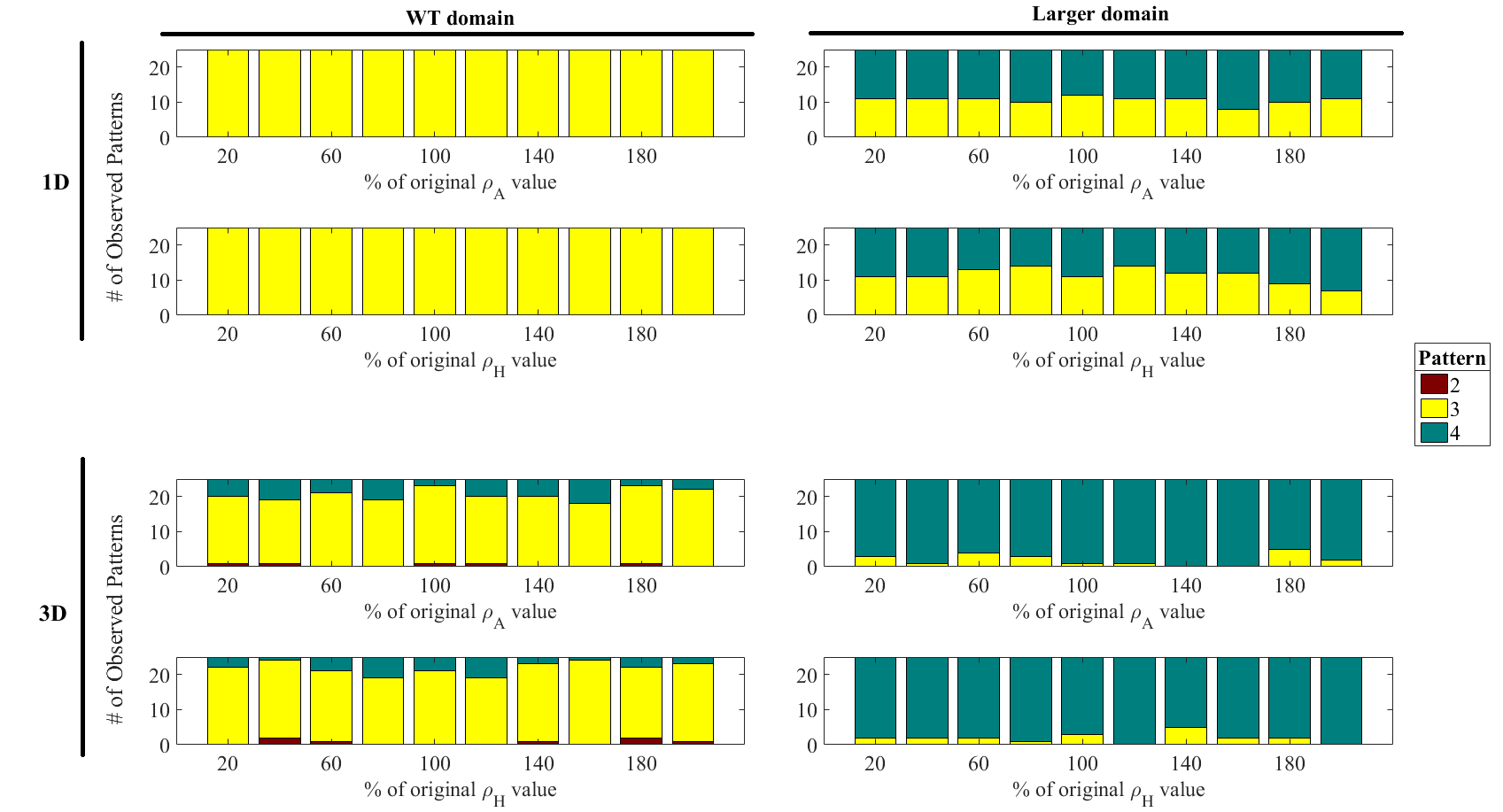

Supplement: S2 Fig — The distribution of patterns produced by changing the value of ρA and ρH from 20% to 200% of the value from Table 1 and simulating each parameter set 25 times. The top row corresponds to the ρA term and the bottom row is ρH. (TIF) [file pcbi.1006800.s002.tif]

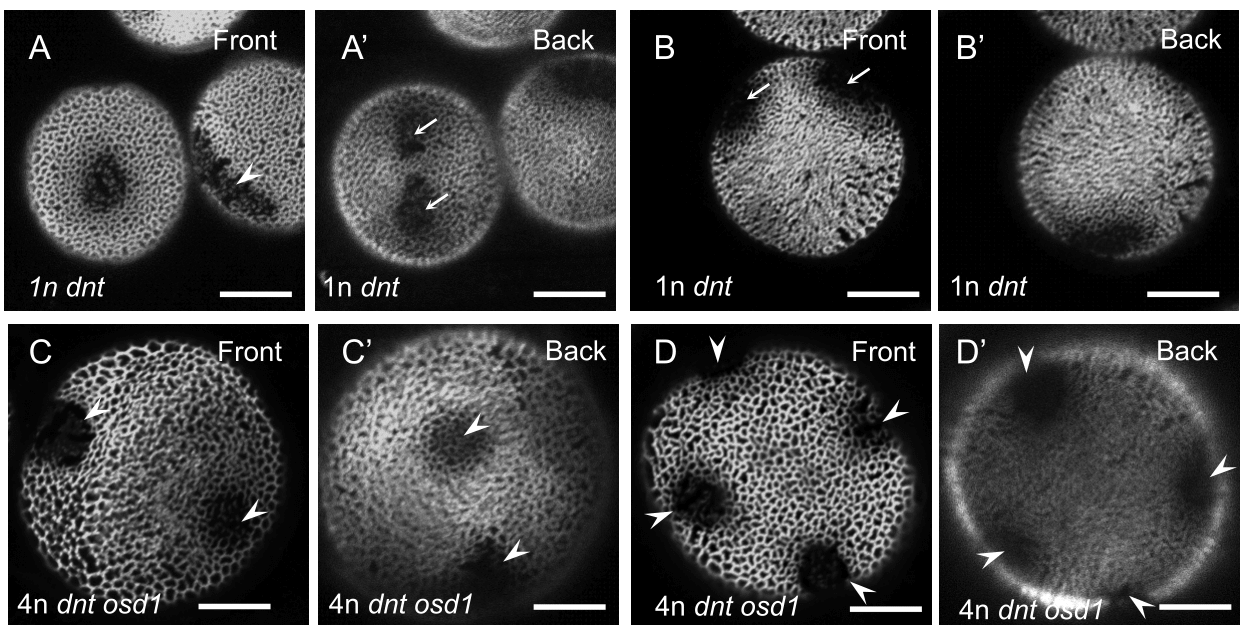

Supplement: S3 Fig — (A-B’) Although many of the haploid dnt pollen grains have two round apertures, pollen with elongated apertures (arrowhead in (A)) and apertures composed of two closely positioned holes (arrows in (A’, B)) is also present. (C-D’) Tetraploid dnt osd1 pollen usually has more than two round apertures. (C, C’) An example of a tetraploid dnt osd1 pollen grain with four round apertures (arrowheads). (D, D’) An example of a tetraploid dnt osd1 pollen grain with eight round apertures (arrowheads). See also S10 Video. Scale bars = 10 μm. (TIF) [file pcbi.1006800.s003.tif]

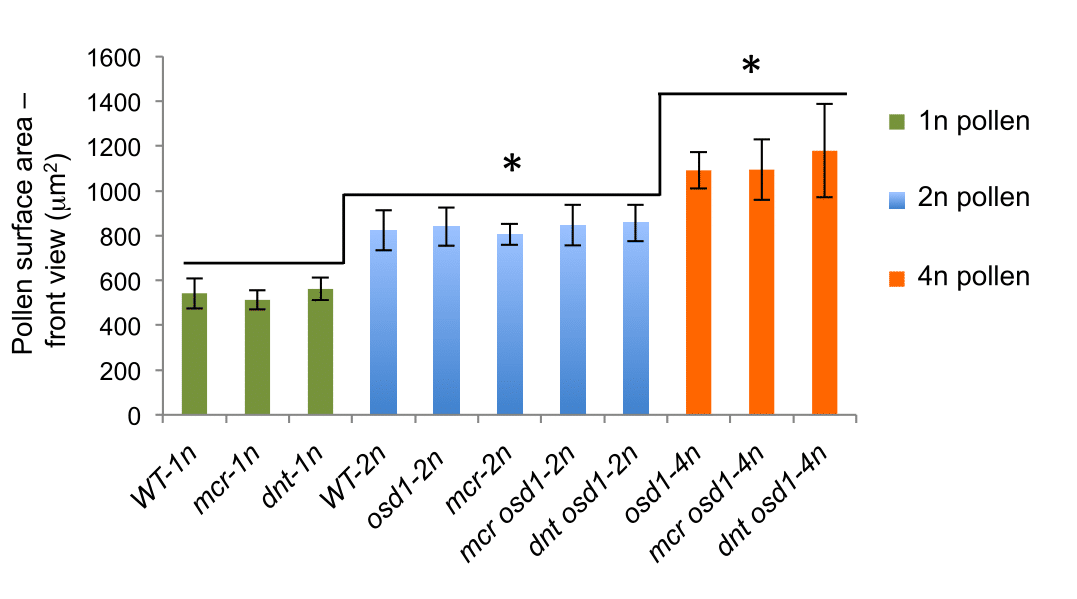

Supplement: S4 Fig — Areas of pollen surface visible in the ‘front view’ images were measured for pollen of the genotypes examined in this study. Data are shown as mean ± SD. Pollen sizes are significantly different between the pollen grains of different ploidy (p-value <0.05, indicated by asterisks) but not between pollen of different genotypes that have the same ploidy. (TIF) [file pcbi.1006800.s004.tif]

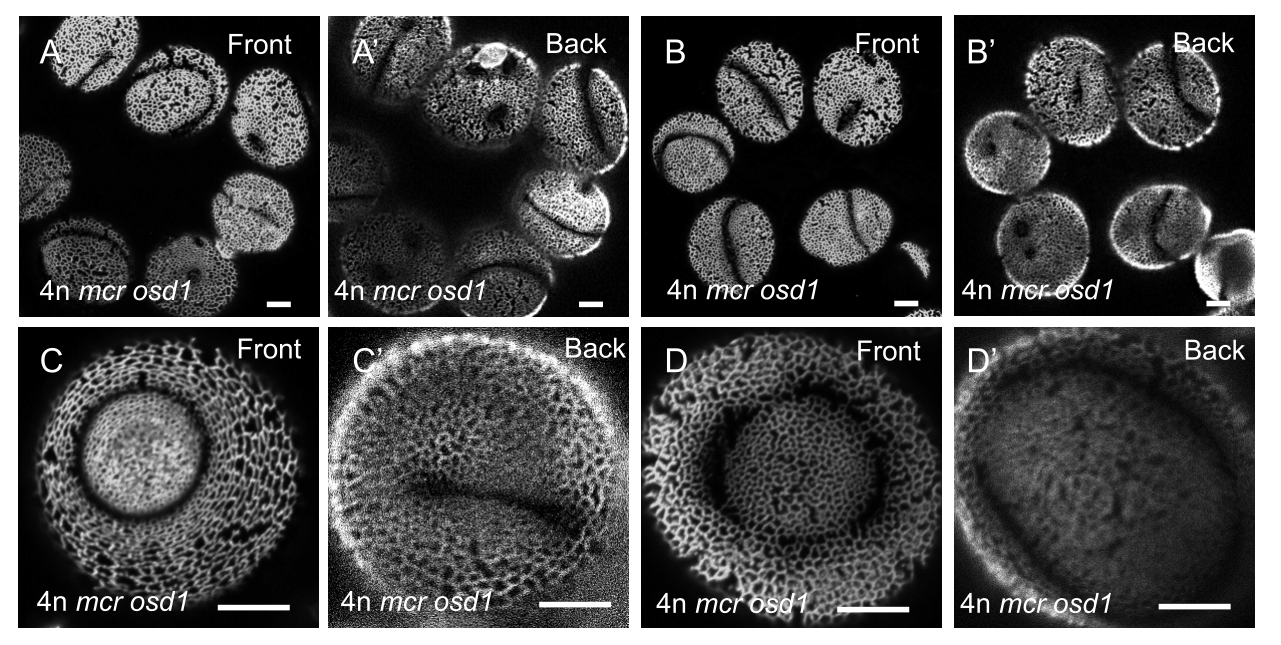

Supplement: S5 Fig — (A-D’) 4n mcr osd1 pollen commonly develops a ring-shaped aperture displaced to one side of the grain and one or two dot-like apertures on the opposite side. Scale bars = 10 μm. (TIF) [file pcbi.1006800.s005.tif]
